# Macrophage-driven immunopathology in pulmonary arterial hypertension: from mechanisms to targeted therapies

**DOI:** 10.3389/fimmu.2025.1721071

**Published:** 2025-12-17

**Authors:** Wenna Xu, Yunlong Shen, Zhengdong Wan, Jiawei Guo

**Affiliations:** 1 Department of Vascular and Endovascular Surgery, The First Affiliated Hospital of Yangtze University, Jingzhou, Hubei, China; 2 Department of Pharmacology, School of Medicine, Yangtze University, Jingzhou, China; 3 School of Medicine, Yangtze University, Jingzhou, China

**Keywords:** epigenetic modifications, macrophage polarization, metabolic reprogramming, pulmonary arterial hypertension, pulmonary vascular remodeling, therapeutic targets

## Abstract

Pulmonary arterial hypertension (PAH) is a progressive vascular disorder characterized by obstructive vascular remodeling driven by the aberrant proliferation of endothelial cells, smooth muscle cells, and adventitial fibroblasts within the small pulmonary arteries. Emerging evidence highlights a pivotal role for macrophage polarization in PAH pathogenesis. In the pulmonary vasculature, macrophages drive local inflammation and fibrosis through M1/M2 polarization, while the inflammatory mediators they release can also alter the systemic immune environment and indirectly influence right ventricular remodeling through the “lung–heart immune axis.” This phenotypic plasticity is tightly governed by hypoxia-induced signaling pathways, metabolic reprogramming, and epigenetic modifications. Elucidating these mechanisms has revealed macrophage polarization and immunometabolic regulation as promising therapeutic targets for PAH. Future investigations focusing on macrophage heterogeneity, single-cell transcriptomics, and precision immunomodulatory strategies are expected to accelerate the development of targeted therapies and improve clinical outcomes in PAH.

## 1 Introduction

According to the latest hemodynamic criteria, pulmonary arterial hypertension (PAH) is defined as a mean pulmonary arterial pressure (mPAP) >20 mmHg at rest, measured by right heart catheterization (1). Unlike other types of pulmonary hypertension caused by left heart disease, lung parenchymal disorders, or chronic thromboembolic factors, the core etiology of PAH lies in the intrinsic remodeling of the pulmonary vasculature itself (2).PAH is a severe cardiopulmonary disorder characterized by pulmonary vascular remodeling and progressive pre-capillary arteriolar obstruction. These structural and functional abnormalities increase pulmonary vascular resistance, drive chronic pressure overload of the right ventricle(RV), and ultimately culminate in right-heart failure, resulting in substantial morbidity and mortality (3). Despite meaningful advances in clinical management, PAH remains incurable and continues to pose a major threat to life. Current therapies predominantly act as vasodilators that rebalance dysregulated vasoactive pathways and relieve symptoms; however, they do not effectively halt or reverse the underlying disease process (4). Consequently, there is an urgent need for mechanistic studies that interrogate the fundamental pathology of PAH and for the development of truly disease-modifying interventions.

Historically, research into PAH pathogenesis focused on vasoconstriction, smooth-muscle proliferation, and fibrosis (5). More recently, immune-inflammatory mechanisms have emerged as central drivers of disease initiation and progression rather than merely accompanying phenomena. Among the immune populations implicated, macrophages—master regulators of innate immunity—play a particularly prominent role (6). Macrophages exhibit remarkable plasticity and undergo functional reprogramming in response to microenvironmental cues, a process termed macrophage polarization (7). Classically activated M1 and alternatively activated M2 macrophages secrete distinct cytokine and growth-factor repertoires that differentially shape chronic inflammation, pulmonary vascular remodeling, and right-ventricular dysfunction (8). As such, macrophages serve as a critical interface linking immune dysregulation to pulmonary vascular pathology. Macrophages in PAH tend to exhibit activation features that promote vascular remodeling, metabolic reprogramming, and angiogenesis. In contrast, in pulmonary hypertension primarily driven by hypoxia or tissue injury, macrophage activation is more likely a secondary response to the hypoxic microenvironment or tissue damage, and their gene expression profiles and functional states may differ from those of macrophages in PAH (9, 10).

This review synthesizes current evidence on the central roles of macrophages in PAH, delineates the regulatory networks that govern their polarization and function, and surveys emerging macrophage-targeted therapeutic strategies. By integrating mechanistic and translational insights, we aim to provide a foundation for precision immunomodulation in PAH and to identify opportunities for the development of disease-modifying therapies.

## 2 Origin of macrophages

In 1968, van Furth and his colleagues first proposed the “mononuclear phagocyte system” theory, which hypothesized that all tissue macrophages are derived from bone marrow monocytic precursors (11). This concept, as a classical paradigm in immunology, profoundly shaped the understanding of macrophage origin and differentiation over the past several decades. However, accumulating evidence now indicates that certain macrophage populations do not depend on monocyte differentiation, or even on adult bone marrow hematopoiesis. These tissue-resident macrophages originate from two successive waves of embryonic precursor colonization. The first wave arises from erythro-myeloid progenitors (EMPs) in the yolk sac, which directly give rise to primitive macrophages in a c-Myb–independent manner and without a monocyte intermediate, ultimately forming microglia. The second wave derives from c-Myb–positive EMPs, which migrate to the fetal liver, differentiate into fetal monocytes, and subsequently give rise to most adult tissue-resident macrophages, including Kupffer cells, alveolar macrophages, and Langerhans cells (3, 4, 12). Embryonically derived macrophages develop into self-renewing resident populations in adulthood, maintaining tissue homeostasis under steady-state conditions. In contrast, bone marrow–derived monocytes are recruited from the peripheral circulation to tissues only during injury, infection, or inflammation, where they differentiate into macrophages to meet pathological demands (13, 14).

Based on their localization and function, pulmonary macrophages can be categorized into alveolar macrophages (AMs) and interstitial macrophages (IMs). These two populations differ in developmental origin: AMs mainly derive from fetal liver progenitors, whereas IMs originate from yolk sac progenitors and can be further supplemented by recruited blood monocytes (15). During the progression of PAH, the homeostasis of tissue-resident macrophages becomes disrupted, while circulating monocytes—particularly those with intrinsic abnormalities identified in patients with idiopathic PAH carrying BMPR2 mutations—are recruited into the pulmonary vasculature, thereby contributing to disease initiation and progression. (16). These peripherally recruited macrophages markedly increase in number and exhibit sustained activation and a pro-inflammatory phenotype, thereby reshaping the local immune microenvironment and driving key pathological processes of PAH, including vascular inflammation and remodeling (17, 18).

The dynamic changes and phenotypic characteristics of these macrophage populations have been validated through single-cell RNA sequencing and flow cytometry (16, 17). This dynamic regulation of macrophage subpopulations not only underlies the immunopathological basis of PAH but also provides a potential entry point for subset-specific macrophage–targeted interventions (6).

## 3 Macrophage polarization

Macrophages, as a functionally diverse class of immune cells, exhibit high plasticity and can adapt to different tissue microenvironments to perform a wide range of functions (7). They are classified according to their activation states into classically activated pro-inflammatory M1 macrophages and alternatively activated anti-inflammatory M2 macrophages (19).

### 3.1 M1 macrophages

M1 macrophages are primarily induced by signals such as bacterial lipopolysaccharide (LPS) and interferon-γ (IFN-γ), which activate transcriptional pathways including signal transducer and activator of transcription 1(STAT1) and NF-κB to initiate and sustain a robust pro-inflammatory response (20). These cells typically express marker molecules such as Cluster of Differentiation 80 (CD80), CD86, inducible nitric oxide synthase (iNOS), and major histocompatibility complex class II (MHC-II), and they secrete abundant pro-inflammatory cytokines, including tumor necrosis factor-α (TNF-α), interleukin-1β (IL-1β), IL-6, and IL-12. In addition, they produce large amounts of nitric oxide (NO) and reactive oxygen species (ROS), thereby effectively eliminating bacteria, viruses, and tumor cells (21, 22).

### 3.2 M2 macrophages

M2 macrophages are mainly polarized under the mediation of IL-4, IL-13, and IL-10 through activation of the STAT6 signaling pathway (23). Their typical markers include CD206, CD163, arginase-1 (Arg1), found in inflammatory zone 1 (Fizz1), and chitinase-like protein 3(Ym1) (24). M2 macrophages secrete anti-inflammatory and tissue repair–related factors such as IL-10, transforming growth factor-β (TGF-β), and vascular endothelial growth factor (VEGF), thereby promoting extracellular matrix(ECM) deposition, angiogenesis, and fibrosis (25).

M2 macrophages are not a homogeneous population but are further subdivided into M2a, M2b, M2c, and M2d subtypes based on their inducing signals and functional characteristics (26).M2a macrophages, driven by IL-4 and IL-13, primarily promote tissue repair and fibrosis by secreting TGF-β and platelet-derived growth factor (PDGF), which stimulate fibroblast proliferation and collagen deposition. Upon activation by immune complexes via Toll-like receptors (TLRs) or IL-1 receptors, M2b macrophages secrete IL-10 and TNF-α, displaying a unique functional profile that combines mild pro-inflammatory activity with immunoregulation. (27, 28).M2c macrophages, induced by IL-10,TGF-β,and glucocorticoids, secrete IL-10 and TGF-β and display potent anti-inflammatory activity, mainly mediating inflammation resolution and tissue remodeling. (29)M2d macrophages, also known as tumor-associated macrophages, are induced by signals such as adenosine and IL-6 and secrete IL-10 and VEGF, thereby significantly promoting angiogenesis and contributing to immune evasion within the tumor microenvironment (30).

**Figure 1:**
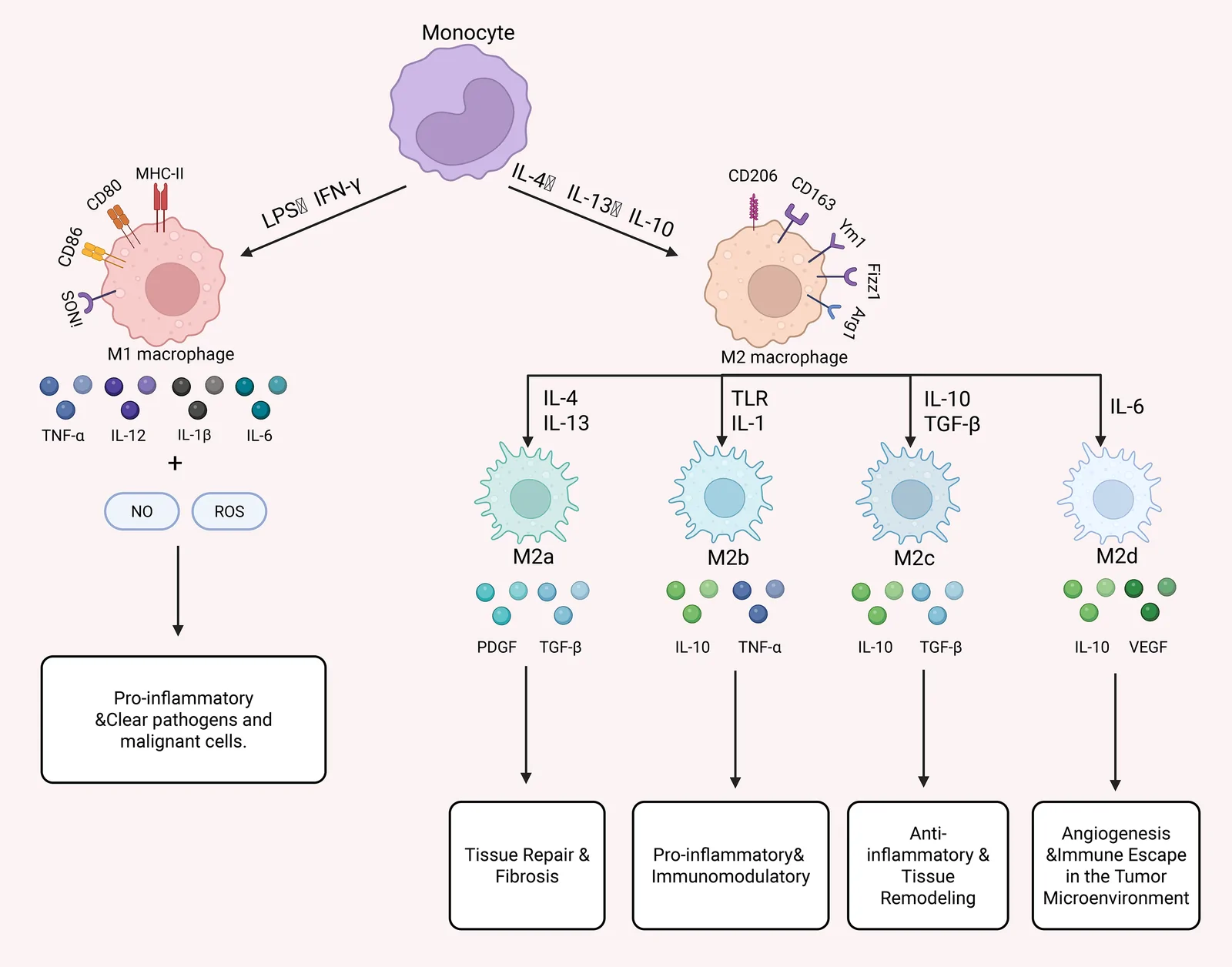
Macrophage polarization and functional specialization.

Macrophages can polarize into either pro-inflammatory M1 or anti-inflammatory M2 phenotypes in response to distinct environmental cues. M1 macrophages, activated by LPS and IFN-γ, are characterized by high expression of CD86, CD80, MHC-II, and iNOS. They secrete high levels of inflammatory mediators such as TNF-α, IL-12, IL-1β, and IL-6, along with ROS and NO, which contribute to pathogen clearance and antitumor defense. Conversely, M2 macrophages, induced by stimuli including IL-4, IL-13, and IL-10, typically upregulate markers like CD206, CD163, Ym1, Fizz1, and Arg1. They are further classified into M2a, M2b, M2c, and M2d subsets, each defined by a unique cytokine profile and specialized roles in immune regulation, tissue repair, and angiogenesis (**Figure 1**).

### 3.3 A new perspective on macrophage heterogeneity: beyond the M1/M2 paradigm

Although the M1/M2 polarization framework provides a foundational model for understanding macrophage functions, its limitations—arising from *in vitro* stimulation models—have become increasingly apparent, as it cannot fully capture the high heterogeneity and dynamic plasticity of macrophages within the complex PAH microenvironment. Recent single-cell RNA sequencing studies have gradually revealed that the macrophage subsets present in the pulmonary vasculature of PAH far exceed the traditional binary classification. For example, Jiang et al. identified APOE^+^ and MARCO^+^ macrophage subsets, as well as dysfunctional GPNMB^+^ populations, highlighting the remarkable diversity of macrophage functional states in PAH (31). A review by Tang et al. further emphasized that single-cell sequencing technologies are systematically reshaping our understanding of macrophage polarization lineages (32). Early scRNA-seq studies in mice and humans also provide supporting evidence; for instance, Mao et al. identified a CX3CR1^+^ macrophage cluster in a hypoxic PAH model, which, although expressing some “M1 marker genes,” does not fully conform to the classical M1 transcriptional profile (33). Taken together, in discussions of PAH pathogenesis, the M1/M2 framework should be regarded as a generalized description of macrophage pro-inflammatory or reparative/remodeling tendencies, rather than a precise definition of their complex *in vivo* states.

## 4 The role of macrophages in PAH

### 4.1 Macrophage polarization and pulmonary vascular inflammation

Extensive infiltration of macrophages around the pulmonary vasculature is a pathological feature observed in both patients with PAH and animal models, as supported by substantial evidence (34). Recent studies have further revealed that both M1 and M2 macrophages play important roles in pulmonary vascular inflammation.

In the monocrotaline (MCT)-induced rat model of PAH, early accumulation of M1 macrophages around the pulmonary vasculature can be observed, accompanied by high expression of inducible iNOS and IL-1β (35). M1 macrophages secrete high levels of pro-inflammatory cytokines. These cytokines induce pulmonary endothelial cells to upregulate the expression of intercellular adhesion molecule-1, vascular cell adhesion molecule-1 (VCAM-1), and E-selectin, thereby enhancing the adhesion and migration of circulating immune cells and driving massive recruitment of monocytes and neutrophils, forming an amplified inflammatory cascade. This role of M1 macrophages has been further confirmed in hypoxia-induced PAH animal models, where they serve as major sources of inflammatory chemokines, particularly C-C Motif Chemokine Ligand 2(CCL2) and C-X-C Motif Chemokine Ligand 10(CXCL10). These chemokines promote continuous infiltration of bone marrow–derived monocytes around the pulmonary vasculature via the C-C Motif Chemokine Receptor 2(CCR2) and C-X-C Motif Chemokine Receptor 3(CXCR3) signaling axes, forming a positive feedback loop within the inflammatory microenvironment (17, 36–39). This sustained inflammatory recruitment is considered one of the initiating events in vascular pathology. Additionally, under the action of caspase-1, IL-18 is converted from its inactive precursor to a biologically active form. By activating the core signaling axis of type I immune responses, it synergistically induces an expression cascade of key effector factors including CXCL10, IL-12,and IFN-γ, thereby enhancing specific chemotaxis and infiltration of lymphocytes into the pulmonary vascular wall and ultimately exacerbating the pathological progression of PAH. (40).Macrophage polarization amplifies inflammation not only through cytokine secretion but also via interactions with other immune cells. M1 macrophages produce cytokines such as IL-12 to drive the differentiation and activation of Th1 and Th17 cells, enhancing the release of pro-inflammatory mediators, activating endothelial cells, and promoting immune cell infiltration, thereby further amplifying the inflammatory response (41).

#### 4.1.1 The role of M2 macrophages in inflammation

M2 macrophages serve as crucial immunomodulators within the inflammatory network. Their M2b and M2c subsets generate IL-10 and TGF-β, effectively curbing excessive M1-type cytokine production while simultaneously promoting the resolution of inflammatory responses. (35). IL-10 exerts its immunosuppressive effects by downregulating the expression of MHC class II molecules and costimulatory molecules on antigen-presenting cells, thereby inhibiting T cell activation and proliferation (42). TGF-β, on the other hand, exerts immunosuppressive functions through multiple mechanisms: it not only directly inhibits T cell activation, proliferation, and the production of pro-inflammatory cytokines, but also promotes the differentiation of regulatory T cells, thus playing a critical role in maintaining immune tolerance and promoting inflammation resolution (43).However, excessive accumulation of M2 macrophages may not fully resolve inflammation; instead, their coupling with Th2 responses can sustain a state of chronic low-grade inflammation (25). The imbalance of macrophage polarization is closely associated with the severity of PAH. Zawia et al. demonstrated using macrophage manipulation models that enhancement of the pro-inflammatory phenotype directly induced PAH-like features in mice, while suppression of inflammatory polarization alleviated vascular lesions to some extent (44).Recent studies have further revealed that in MCT- or hypoxia-induced rat models of PAH, macrophage infiltration and associated inflammatory responses can also be observed in the RV (45). Therefore, inflammation in PAH may represent a systemic process that extends beyond the lungs, also affecting the heart and leading to similar pathophysiological alterations. The finding of macrophage infiltration in the heart is particularly significant, as right ventricular (RV) dysfunction and failure are major determinants of prognosis in PAH patients.

### 4.2 Macrophage polarization and pulmonary vascular remodeling

The polarization state and dynamic evolution of macrophages constitute the core immune mechanism driving pulmonary vascular remodeling in PAH (46). Sushil Kumar et al. found that during the early acute inflammatory phase (days 1–3), the predominant subset is MHC-II^hi CCR2^+ IMs, whose transcriptomic profile is enriched in classical pro-inflammatory pathways such as IFN-γ, IL-2, and IL-6 signaling, exhibiting a pronounced M1-like phenotype that serves as the main effector population mediating early inflammatory responses. In contrast, during the prolonged hypoxia-induced remodeling phase (days 7–21), the TLF^+ VCAM-1^hi IM subset becomes dominant, with functions shifting toward chemokine production, dysregulated tissue repair, and complement pathway activation—features characteristic of an M2-like phenotype—representing the key cell population driving late-stage vascular pathological remodeling (47). In the early stages of the disease, M2 macrophages exhibit anti-inflammatory properties; however, under persistent stimulation, they transition into an “over-repair” phenotype.

#### 4.2.1 The role of M1 macrophages in pulmonary vascular remodeling

M1 macrophages promote endothelial injury and ECM remodeling by secreting pro-damaging factors such as IL-1β, TNF-α, ROS, and matrix metalloproteinases (MMPs), thereby disrupting endothelial homeostasis and altering ECM composition. These effects impair endothelial adhesion and barrier integrity, disturb cell–matrix adhesion balance, and create a microenvironment conducive to endothelial-mesenchymal transition (EndMT) (48).The underlying mechanisms involve two main aspects: (1) direct activation of signaling pathways such as TGF-β/Smad and MAPK, leading to the loss of endothelial-specific markers including vascular endothelial cadherin and platelet endothelial cell adhesion molecule-1, while inducing the expression of mesenchymal proteins such as alpha-smooth muscle actin and fibronectin (49); and (2) MMP-mediated ECM degradation and ROS-induced oxidative stress, which synergistically amplify pro-trans differentiation signaling (19).In PAH patients and animal models, M1 macrophages promote the phenotypic transition of vascular smooth muscle cells (VSMCs) toward proliferative and migratory states by upregulating MMP-1 and MMP-10 expression. Notably, the STAT1 signaling pathway is involved in regulating hypoxia-induced MMP-10 expression but does not affect MMP-1 expression levels. These findings highlight the pivotal role of macrophage-derived MMP-10 in vascular remodeling during PAH and suggest that circulating MMP-10 levels may serve as a potential biomarker and therapeutic target for PAH (50).In recent years, single-cell studies have confirmed the presence of prominent EndMT-related gene signatures within the vascular endothelial cells of PAH patients, which, together with macrophage-derived signaling, form an interconnected molecular network driving vascular remodeling (51).

#### 4.2.2 The role of M2 macrophages in pulmonary vascular remodeling

Repair-associated macrophages such as M2a and M2d act as key mediators in the tissue repair process. They drive fibrosis and angiogenesis by secreting large amounts of core growth factors, including TGF-β, PDGF, and VEGF (52).

M2 macrophages participate in pathological processes primarily through the secretion of large amounts of TGF-β, which in turn further promotes macrophage polarization toward the M2 phenotype, forming a positive feedback loop. TGF-β regulates cell proliferation, phenotypic remodeling, metabolism, and immune responses, thereby influencing tissue homeostasis and repair. It also effectively modulates fibroblast activation, epithelial–mesenchymal transition (EMT), and ECM remodeling (53). In PAH, overactivation of the SMAD2/3 signaling pathway in pulmonary arterial endothelial cells and smooth muscle cells, along with the suppression of SMAD1/5/8 signaling, jointly drives pulmonary vascular structural remodeling (54, 55).

This mechanism has been confirmed in MCT-induced experimental models of PAH: pulmonary TGF-β signaling is markedly upregulated, whereas blocking TGF-β receptor type I to inhibit excessive SMAD2/3 activation significantly attenuates vascular remodeling. This is manifested by the induction of abnormal apoptosis, inhibition of adventitial cell proliferation, and reduction of ECM degradation, providing direct evidence for targeting this pathway as a therapeutic strategy for PAH (56). Elevated TGF-β1 expression in lung tissues of PAH patients correlates with disease severity (57).

Macrophage-derived PDGF serves as a central factor driving vascular remodeling, with PDGF-B playing a particularly critical role. PDGF-B acts mainly through activation of platelet-derived growth factor receptor beta (PDGFR-β), which is predominantly expressed on pulmonary arterial smooth muscle cells (PASMCs). PDGFR-β activation further triggers multiple downstream signaling pathways, including MAPK and AKT–mTOR, thereby strongly promoting PASMC proliferation and migration. This macrophage-driven signaling axis has been shown to induce vascularization in mouse models and to promote phenotype switching in human PASMCs when exposed to macrophage-conditioned medium. These findings are consistent with clinical evidence showing upregulation of PDGFR-β expression in lung tissues of PAH patients (58, 59).

VEGF is a central factor driving pathological angiogenesis. It plays an indispensable role in microvascular network formation by promoting endothelial cell proliferation, migration, and tube formation (59). However, under conditions of persistent inflammation and high-stress pathological microenvironments, VEGF-induced neovessels often exhibit abnormal structure and immature function. Such unstable angiogenesis not only fails to improve tissue perfusion effectively but may also exacerbate perivascular thickening and remodeling, ultimately accelerating PAH progression (60). Collectively, these mechanisms drive medial thickening, smooth muscle hyperplasia, and fibrosis in pulmonary vessels.

#### 4.2.3 Irreversible vascular lesions

The long-term imbalance or dynamic switching between M1 and M2 macrophage phenotypes constitutes the core pathological basis of pulmonary vascular remodeling in PAH. As a result, the acute, injury-driven inflammatory response and the chronic, repair-oriented fibrotic process form a vicious cycle that jointly drives vascular structures toward irreversible occlusive lesions. The pathological process features aberrant hyperproliferation of vascular smooth muscle components accompanied by excessive ECM deposition, along with permanent endothelial dysfunction and luminal narrowing—characteristics that precisely mirror the irreversible vascular lesions observed in clinical settings. (8, 44, 61).

#### 4.2.4 Mediating right heart failure

**Figure 2:**
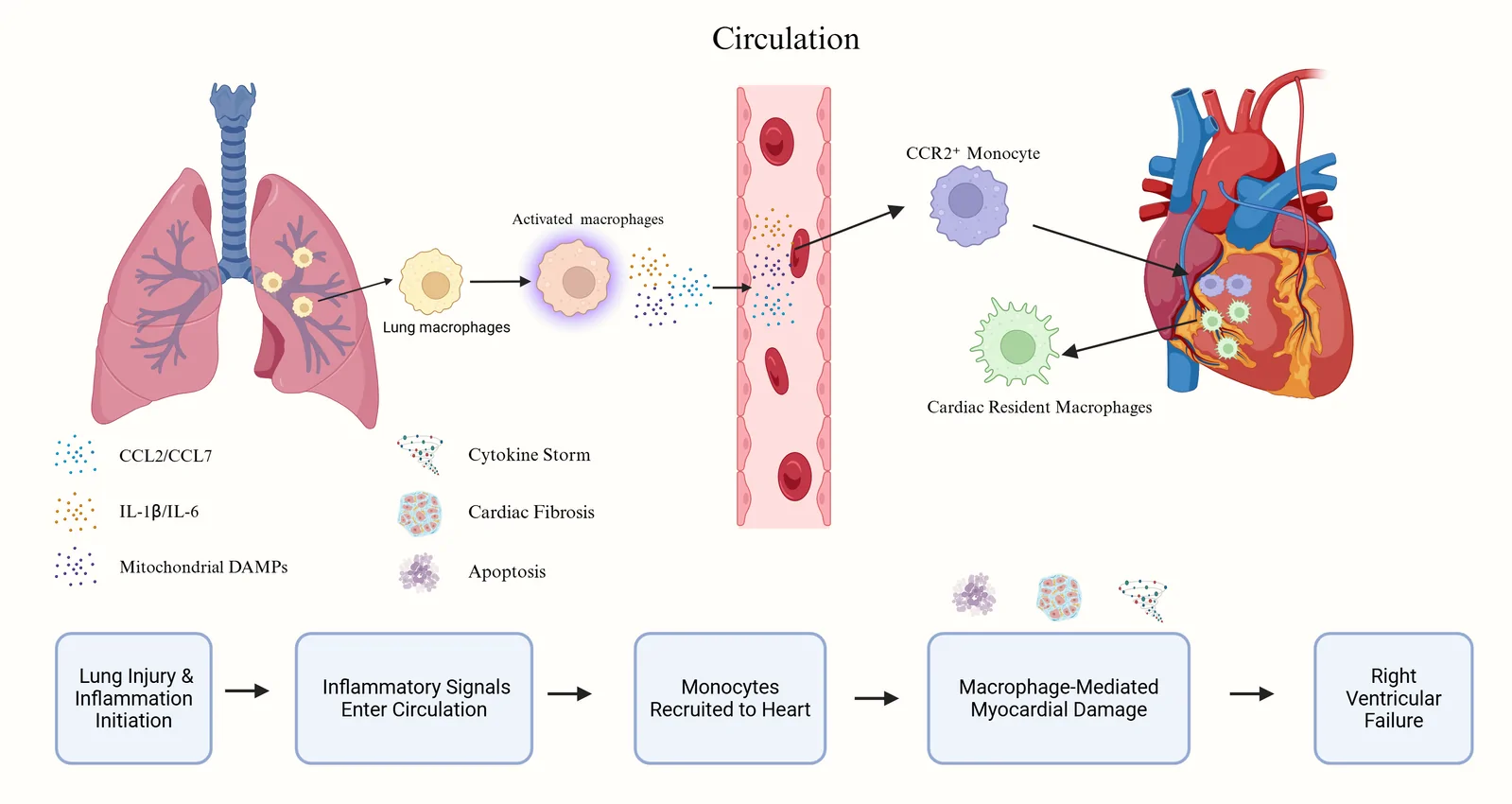
The lung-heart axis in pulmonary arterial hypertension.

Beyond local pulmonary vascular pathology, increasing evidence indicates that PAH is a systemic disease involving immune remodeling of both the lungs and the heart (62). Activation of perivascular macrophages in the lungs, through amplification of inflammation, chemokine release, and extracellular matrix remodeling, can induce systemic shifts in the circulating monocyte–macrophage compartment, thereby altering the immune microenvironment of the RV (63). These lung-derived inflammatory signals can drive directed migration of peripheral monocytes into the RV, promoting a shift of the RV macrophage population toward pro-inflammatory and pro-fibrotic phenotypes (64), which accelerates myocardial interstitial deposition, mitochondrial stress, and cardiomyocyte structural damage. This cross-organ inflammation–remodeling interaction, referred to as the “lung–heart axis,” suggests that pulmonary vascular inflammation and RV failure in PAH are not isolated processes, but are coupled through the immune system and circulating signals, with the overall coordination imbalance ultimately determining disease progression and prognosis (**Figure 2**).

Pulmonary vascular remodeling and pulmonary hypertension lead to a sustained increase in RV afterload, serving as the initiating factor that triggers the progression toward right heart failure (65). In the early stages of the disease, the RV adapts to the abnormally elevated pulmonary vascular resistance through compensatory hypertrophy, a process that increases myocardial mass to maintain ventricular wall stress at a relatively normal level and preserve cardiac output (66). However, this compensatory capacity has a physiological limit.

Persistent pressure overload induces a cascade of pathological events: on one hand, sustained high pressure leads to insufficient myocardial perfusion—particularly in the subendocardial regions prone to ischemia—and strongly activates the renin–angiotensin–aldosterone system and the sympathetic nervous system, resulting in sodium and water retention, peripheral vasoconstriction, and myocardial fibrosis (65, 67). On the other hand, abnormal mechanical stress and neurohormonal activation together promote the transition of cardiomyocytes from compensatory hypertrophy to apoptosis and necrosis, while concurrently activating cardiac fibroblasts, causing excessive ECM deposition and interstitial fibrosis (68). Notably, immune and inflammatory mechanisms are central drivers of RV remodeling and failure. During the progression of PAH-induced RV dysfunction, specific subpopulations of macrophages derived from circulation have been shown to exert important pathological effects. Studies in multiple PAH animal models—including those induced by hypoxia, schistosomiasis infection, and SU5416 combined with hypoxia—demonstrate that although the total number of RV macrophages remains unchanged, the proportion of specific subsets significantly increases. Parabiosis experiments further confirmed that these macrophages are recruited from the circulation rather than generated by local cardiac proliferation (69).Activated macrophages, together with infiltrating T lymphocytes, accumulate in the myocardium and release pro-inflammatory cytokines, thereby exacerbating myocardial injury and structural remodeling (70). Another study revealed a more specific mechanism underlying RV failure: under PAH conditions, circulating CCR2^+^ macrophages selectively infiltrate the RV and express high levels of the NOD-, LRR- and pyrin domain-containing protein 3 (NLRP3) inflammasome, leading to mitochondrial dysfunction in cardiomyocytes. This phenomenon has been validated in both MCT and Su/Hx rat models as well as in cardiac tissue samples from PAH patients. Importantly, this macrophage-mediated inflammatory activation exhibits clear RV specificity, as no comparable pathological changes were observed in the left ventricle or in models of isolated pressure overload (45).

Together, these pathological processes interact to form a vicious cycle, ultimately driving the progression of the RV from compensatory hypertrophy to irreversible decompensated dilation, loss of contractile function, and extensive fibrosis—making right heart failure the key determinant of prognosis in patients with PAH (66).

This schematic illustrates the core mechanism in the pathogenesis of pulmonary arterial hypertension: Pulmonary vascular injury activates lung macrophages, releasing inflammatory mediators such as CCL2/CCL7, IL-1β/IL-6, and mitochondrial DAMPs into the circulatory system. These inflammatory signals mediate the recruitment of monocytes to the heart via the CCL2-CCR2 axis, where they differentiate into macrophages. The recruited macrophages, together with resident cardiac macrophages, become activated and drive right heart failure through mechanisms including the induction of a cytokine storm, promotion of myocardial fibrosis, and triggering of cardiomyocyte apoptosis.

## 5 Mechanisms of macrophages in PAH

The functional abnormalities of macrophages in PAH do not occur in isolation but rather result from the precise regulation of a complex molecular network. The following sections will systematically elaborate on the key upstream mechanisms driving macrophage phenotypic and functional transitions, ranging from classical hypoxic signaling to cutting-edge metabolic and epigenetic regulation.

### 5.1 Hypoxia

The hypoxia–HIF axis serves as a central bridge linking low-oxygen conditions to immune–metabolic dysregulation in the pathogenesis of PAH.

Hypoxia-inducible factors (HIFs), composed of an oxygen-sensitive α subunit and a constitutively expressed β subunit, act as master regulators that enable cells to sense and respond to changes in oxygen availability (71). Under normoxic conditions, the HIF-α subunit undergoes hydroxylation by prolyl hydroxylase domain-containing proteins, which allows its recognition and degradation by the von Hippel–Lindau protein. In contrast, under hypoxic conditions, this hydroxylation process is inhibited, leading to the stabilization of HIF-α, which then translocates into the nucleus, dimerizes with HIF-β, and forms an active transcriptional complex. This complex initiates a gene expression program that adapts to hypoxia, regulating angiogenesis, metabolism, and cell proliferation (72).

Multiple clinical studies have demonstrated that both HIF-1α and HIF-2α are broadly upregulated in the lungs and circulation of PAH patients, primarily originating from pulmonary arterial endothelial cells and smooth muscle cells. Activated HIFs promote vascular remodeling, inflammation, and progenitor cell recruitment through the regulation of downstream target genes, collectively driving disease progression (73). In chronic hypoxia–induced PAH mouse models, myeloid cell–specific deletion of HIF-1α reduces pulmonary macrophage infiltration and markedly attenuates vascular remodeling and PAH phenotypes, suggesting that macrophage HIF-1α plays a pro-pathogenic role in hypoxia-driven vascular pathology (74). In addition to acting directly through the HIF signaling pathway, the hypoxia-induced pulmonary vascular microenvironment can also promote complex macrophage phenotypic reprogramming via a paracrine mechanism through the secretion of cytokines. Further studies have shown that in the hypoxic PAH calf model, IL-6 released from pulmonary artery explants activates the STAT3–C/EBPβ axis rather than the classical IL-4/IL-13–STAT6 pathway in macrophages, thereby inducing hallmark molecules such as Arg1 and giving rise to a disease-associated, non-classical alternatively activated state (75).

More broadly, substantial evidence indicates that HIF-2α is a key transcription factor promoting M2 macrophage polarization, capable of directly binding to and activating the promoters of classical M2 genes such as Arg1 and IL-10 (76). However, within the specific pathological context of PAH, most studies on HIF-2α have focused on endothelial and smooth muscle cells. It has been demonstrated that endothelial HIF-2α regulates the expression of factors such as CXCL12 and endothelin-1, playing a central role in mediating vascular construction and remodeling (77, 78). Conversely, the role of macrophage-specific HIF-2α in PAH remains less well understood, with current evidence being largely indirect. The precise mechanisms through which HIF-2α functions in macrophages during PAH require further elucidation.

### 5.2 Metabolic reprogramming

During the pathogenic progression of PAH, metabolic reprogramming serves as the core pathological mechanism driving its self-perpetuating vicious cycle. This metabolic alteration is ubiquitously present among multiple cellular populations within the pulmonary vascular wall, including macrophages, fibroblasts, and endothelial cells—where their dysregulated metabolic states engage in mutual reinforcement through synergistic interactions, collectively culminating in progressive deterioration of pulmonary vascular structure and function.

**Figure 3:**
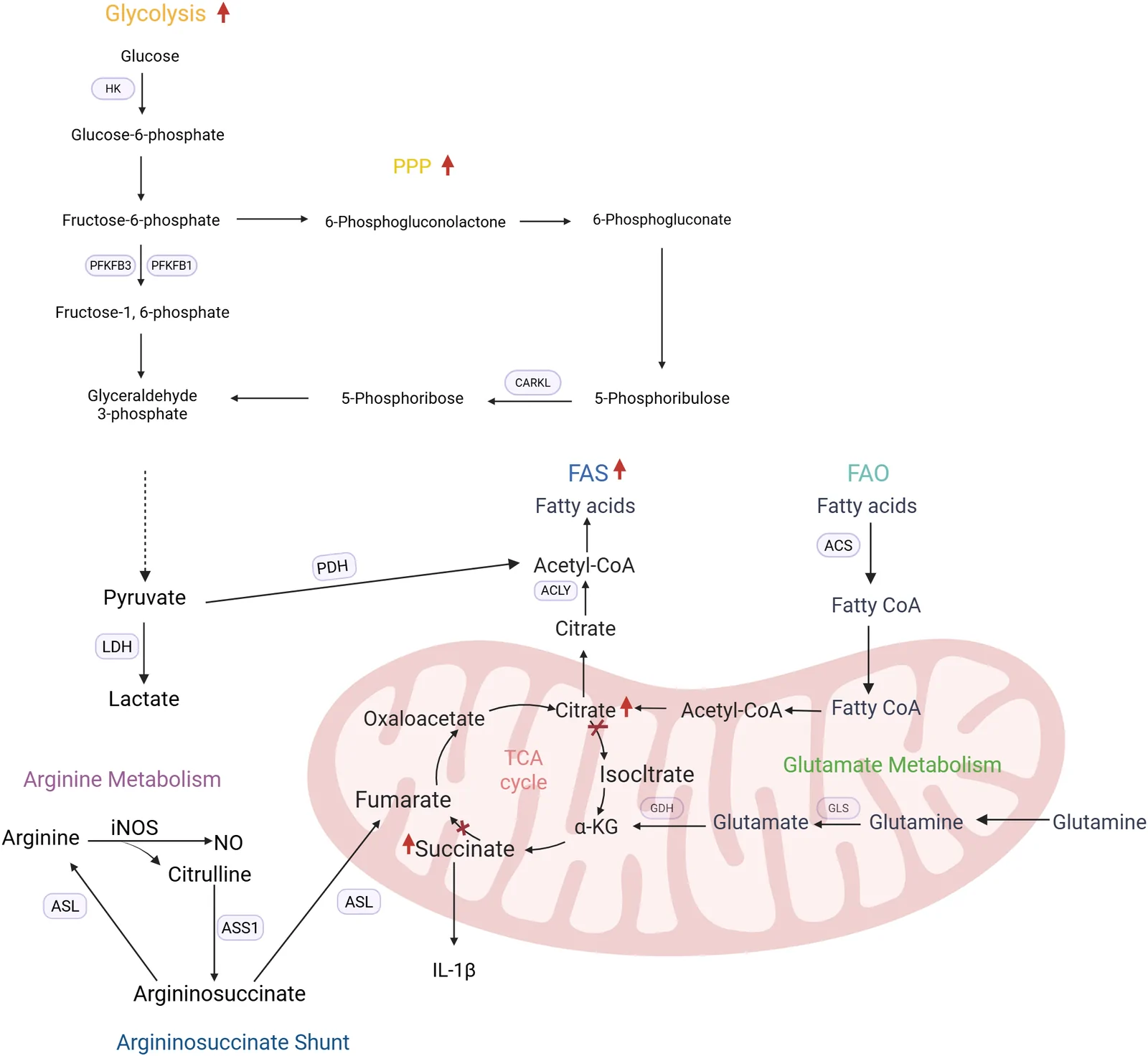
Metabolic characteristics of M1 macrophages.

**Figure 4:**
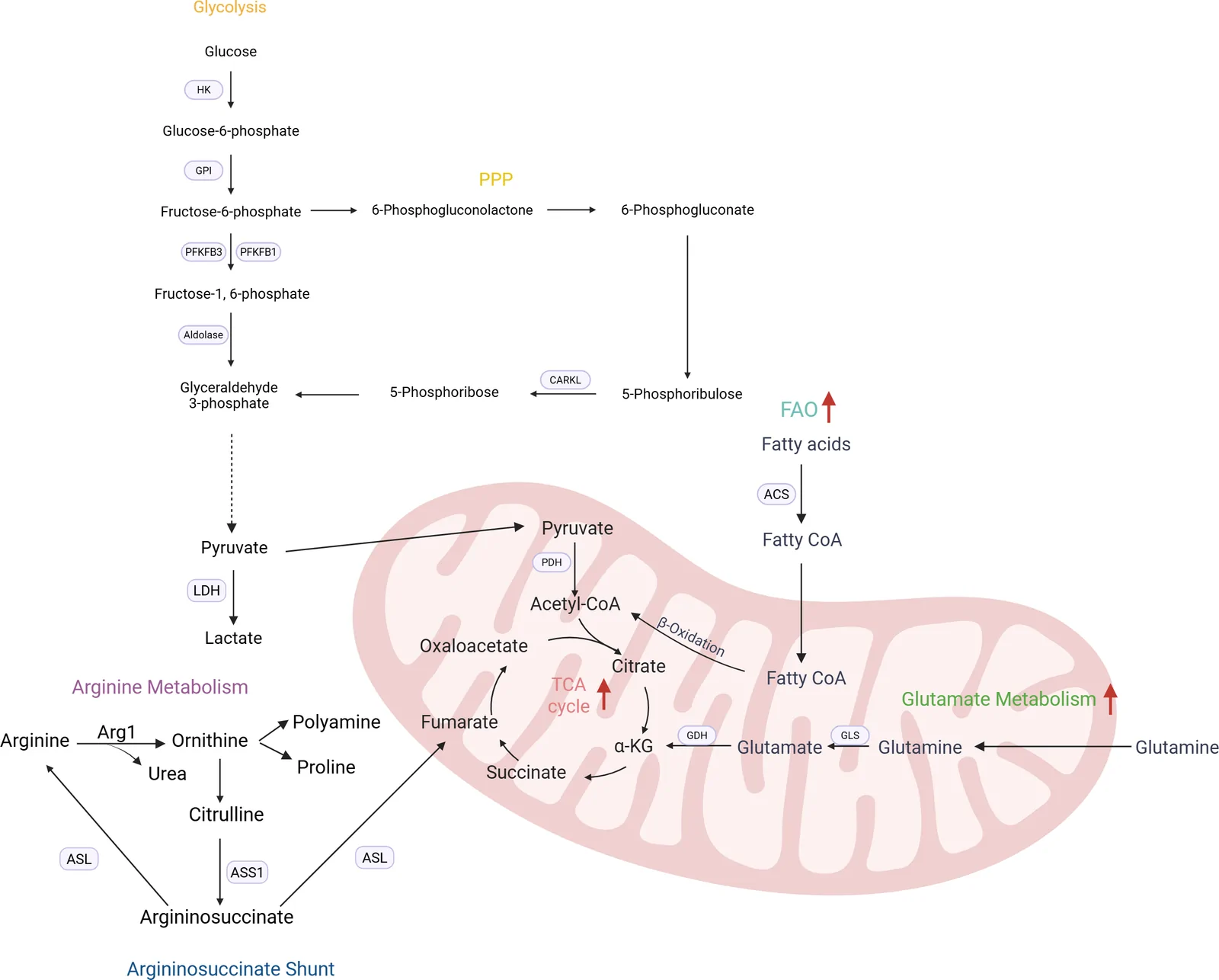
Metabolic characteristics of M2 macrophages.

The polarization of macrophages is accompanied by profound metabolic reprogramming, which endows them with distinct functional characteristics. M1 macrophages are primarily characterized by enhanced glycolysis, pentose phosphate pathway activity, and fatty acid synthesis, with a disrupted tricarboxylic acid cycle leading to the accumulation of metabolites such as succinate and citrate (79) (**Figure 3**). In contrast, M2 macrophages sustain oxidative metabolism and exert anti-inflammatory and reparative functions through increased fatty acid oxidation, a functional tricarboxylic acid cycle, and elevated glutamine metabolism (80, 81) (**Figure 4**). In pulmonary macrophages from patients with PAH as well as in SU5416 + Hypoxia (Su/Hx) rats and hypoxic mice, consistent upregulation of key glycolytic molecules has been observed, indicating the widespread presence of metabolic reprogramming. Myeloid cell–specific deletion of the key glycolytic enzyme 6-Phosphofructo-2-kinase significantly alleviates PAH phenotypes in mice and reduces levels of growth factors and pro-inflammatory cytokines, clearly demonstrating the critical pathogenic role of glycolysis in PAH. Mechanistic studies further reveal that this pronounced glycolytic upregulation is closely associated with pro-inflammatory M1 macrophage polarization (82). Additionally, van L. Brittain et al. reported that in MCT-induced PAH rat models, fatty acid synthesis in M1 macrophages is markedly enhanced. Specifically, lipid metabolism in M1 macrophages within lung tissue and the RV is significantly dysregulated: Fatty acid synthase shows elevated expression along with heightened activity in lung tissue, while RV M1 macrophages exhibit elevated CD36-mediated fatty acid uptake, ultimately leading to intracellular fatty acid accumulation and impaired oxidation (83).On the other hand, in the RV tissue of MCT-induced PAH rats, elevated levels of recombinant solute carrier family 1 member 5, glutamine, and glutamate indicate enhanced glutamine metabolism. Studies have shown that the glutamine antagonist DON improves cardiac function by restoring glucose oxidation, a mechanism consistent with the oxidative phosphorylation–preferred metabolic profile of M2 macrophages (84).

Key metabolic intermediates such as citrate, arginine, and lactate also participate in the regulation of macrophage polarization. Among these, arginine accumulation serves as a critical metabolic hub for macrophage polarization: M1 macrophages highly express inducible nitric iNOS to convert arginine into NO and citrulline. NO not only confers antimicrobial activity but also maintains the epigenetic state, preventing repolarization toward the M2 phenotype (85). In contrast, M2 macrophages highly express Arg1 to hydrolyze arginine into urea and ornithine, the latter of which is further metabolized into polyamines and proline that promote tissue repair (86). These two metabolic pathways compete for the same substrate and exert functionally antagonistic effects, forming a key molecular switch that determines macrophage polarization and immune function. Clinical studies using stable isotope tracing have further revealed that PAH patients with high arginase activity exhibit markedly increased arginine flux accompanied by impaired NO synthesis and lack a compensatory endogenous arginine production mechanism. This indicates that elevated arginase activity is a critical factor leading to dysregulated arginine metabolism and impaired NO synthesis in PAH patients, and inter-individual differences in arginase activity may influence disease phenotype and therapeutic response (87).Meanwhile, citrate accumulates in M1 macrophages and is cleaved by ATP–citrate lyase to generate acetyl-CoA, driving inflammatory lipid synthesis, NADPH production, and histone acetylation, thereby amplifying the expression of pro-inflammatory cytokines such as IL-1β (88, 89). Notably, ATP–citrate lyase has been shown in pulmonary vascular diseases, including PAH, to regulate the glycolysis–lipogenesis metabolic axis and GCN5-mediated epigenetic mechanisms, promoting proliferation and migration of vascular smooth muscle cells. This has been validated using clinical samples, multiple animal models, and ex vivo human models, and ATP–citrate lyase inhibitors effectively attenuate vascular remodeling (90). However, the impact of citrate accumulation specifically in macrophages under PAH conditions remains to be investigated. Lactate, as the end product of glycolysis, accumulates under hypoxic or inflammatory conditions and induces histone lactylation modifications such as H3K18la and H4K5la. Studies indicate that this epigenetic modification is temporally dynamic: in the early phase, it activates M1 inflammatory genes to promote immune responses, whereas in the later phase, it cooperates with p53 and the histone acetyltransferase p300 to activate transcription of reparative genes, driving macrophage polarization from M1 to M2 (91, 92). Although histone lactylation has not yet been directly studied in PAH, given that hypoxia is a central pathological factor in PAH and that significant lactate accumulation and M2 macrophage polarization are observed in PAH patients, histone lactylation may contribute to PAH pathogenesis through these mechanisms, promoting pulmonary vascular remodeling and disease progression.

M1 macrophages undergo metabolic reprogramming towards glycolysis, fatty acid synthesis (FAS), and the pentose phosphate pathway (PPP). A hallmark of this reprogramming is the disruption of the tricarboxylic acid (TCA) cycle at two key points, leading to the accumulation of specific metabolites such as succinate, citrate, and itaconate. The argininosuccinate shunt serves as a critical compensatory mechanism for the disrupted TCA cycle, reclaiming citrulline produced by iNOS to regenerate arginine and replenishing the TCA cycle with fumarate. Relevant enzymes include: Hexokinase (HK); 6-Phosphofructo-2-kinase/Fructose-2,6-bisphosphatase 3 (PFKFB3); Carbohydrate Kinase-Like Protein (CARKL); Lactate Dehydrogenase (LDH); Pyruvate Dehydrogenase (PDH); 6-Phosphofructo-2-kinase/Fructose-2,6-bisphosphatase 1 (PFKFB1); α-Ketoglutarate (α-KG); Glutamate Dehydrogenase (GDH); Glutaminase (GLS); ATP-Citrate Lyase (ACLY); Acyl-CoA Synthetase (ACS); Argininosuccinate Synthase 1 (ASS1); Argininosuccinate Lyase (ASL).

M2 macrophages primarily rely on enhanced fatty acid oxidation (FAO), an intact and active tricarboxylic acid (TCA) cycle, and upregulated glutamine metabolism to maintain their oxidative metabolic homeostasis and immunoregulatory functions. Arginine metabolism can be connected to the TCA cycle via the argininosuccinate shunt. These cells highly express Arg1, which hydrolyzes arginine into urea and ornithine; the latter subsequently generates polyamines and proline to promote tissue repair. Relevant enzymes and metabolites include: Glutamate Dehydrogenase (GDH); Glutaminase (GLS); Hexokinase (HK); Glucose-6-Phosphate Isomerase (GPI); 6-Phosphofructo-2-kinase/fructose-2,6-bisphosphatase 3 (PFKFB3); 6-Phosphofructo-2-kinase/fructose-2,6-bisphosphatase 1 (PFKFB1); Lactate Dehydrogenase (LDH); Pyruvate Dehydrogenase (PDH); Carbohydrate Kinase-Like Protein (CARKL); α-Ketoglutarate (α-KG); Acyl-CoA Synthetase (ACS); Argininosuccinate Synthase 1 (ASS1); Argininosuccinate Lyase (ASL).

### 5.3 Intracellular signaling pathway regulation

#### 5.3.1 NF-κB

The NF-κB signaling pathway, a pivotal determinant in macrophage polarization, plays a critical role in the pathogenesis of PAH. Under resting conditions, NF-κB is retained in the cytoplasm through binding to inhibitor of kappa B (IκB). In response to PAH-related pathological stimuli such as hypoxia and inflammatory factors, the IκB kinase complex is activated, mediating IκB phosphorylation and degradation. Pulmonary vascular remodeling is driven by a NF-κB -dependent signaling axis, which is initiated by its nuclear translocation and subsequent pro-inflammatory gene activation. (93). This mechanism has been validated at multiple levels: histological analyses of clinical tissues show significantly increased NF-κB activity in both the pulmonary arteriolar lesions and AMs of PAH patients (94); single-cell sequencing further confirms sustained activation of this pathway in MCT- and Su/Hx-induced rat models (95).

The role of the TLR–NF-κB axis in macrophage M1 polarization was first systematically elucidated in general immunology research: TLR4 activates NF-κB through the MyD88-dependent pathway, thereby inducing the expression of TNF-α, IL-1β, and IL-6 (96, 97); TLR2 and TLR3 promote proinflammatory cytokine production via the NF-κB/MAPK and TRIF pathways, respectively (98, 99). In addition, the noncanonical NF-κB pathway has also been shown to enhance the transcriptional activity of inflammatory genes (100).

Although most of these studies were not conducted in PAH models, the signaling mechanisms they reveal are supported by corresponding evidence in PAH. In experimental PAH, NF-κB activation is closely associated with elevated IL-1β, IL-6, and TNF-α levels in lung tissues and circulation, and it promotes macrophage polarization toward the M1 phenotype (101). More importantly, in patients with PAH, the mRNA expression of TLR4 and NF-κB in peripheral blood mononuclear cells is significantly upregulated and positively correlated with PASP, suggesting that these canonical inflammatory pathways also possess clinical relevance in PAH (102). Therefore, despite much of the mechanistic understanding originating from general immunology, current evidence supports a critical role for TLR–NF-κB signaling in macrophage-driven inflammatory responses in PAH.

The NF-κB signaling pathway integrates multiple pathological signals through both canonical and non-canonical routes, driving M1 macrophage polarization and pro-inflammatory cytokine release, and playing a central regulatory role in pulmonary vascular inflammation and structural remodeling in PAH. Notably, the NF-κB pathway may also participate in the regulation of M2 macrophage polarization through crosstalk with pathways such as STAT3 (103), although the precise interactions remain to be fully elucidated.

#### 5.3.2 JAK/STAT1

When IFN-γ binds to its cell surface receptor, it activates Janus kinase (JAK), which in turn induces phosphorylation of STAT1. Phosphorylated STAT1 forms dimers and translocates into the nucleus, where it drives the expression of key transcription factors such as interferon regulatory factor 1 and interferon regulatory factor 5, ultimately upregulating M1 macrophage–specific genes including inducible iNOS and MHC-II molecules (19, 104). Significant activation of the STAT1–IFN pathway has been observed in monocytes/macrophages derived from PAH patients, providing direct transcriptomic evidence supporting the clinical relevance of this pathway in human PAH (16).

#### 5.3.3 PI3K/Akt/mTOR

Chao Tang et al. demonstrated that in the MCT-induced PAH model, the inflammatory response evolves in distinct stages: an early acute phase dominated by TNF-α and IL-1β, followed by a chronic phase with concurrent upregulation of IL-6, IL-12, Arg1, and IL-10. Meanwhile, the M1/M2 macrophage ratio remains elevated, and sustained activation of the PI3K/Akt pathway is closely associated with the pro-inflammatory state (105).

Further studies have shown that the PI3K/Akt/mTOR signaling pathway, as a central regulatory hub of cellular metabolism, plays a critical role in the pathogenesis of PAH (106). Activation of this pathway begins with upstream PI3K catalyzing the production of the second messenger PIP3, which in turn activates the central regulator Akt. Akt phosphorylates downstream key effector mTOR, driving the formation of mTORC1/2 complexes and ultimately regulating core metabolic processes such as protein synthesis and autophagy (107). In Su/Hx-induced PAH models and human PASMCs exposed to hypoxia, DNA topoisomerase IIα expression is markedly upregulated, promoting abnormal cell proliferation and migration through activation of the PI3K/Akt/mTOR pathway, and driving inflammatory cytokine release along with the production of ROS (108).

In terms of immunometabolic regulation, multiple studies in tumor and inflammatory models have shown that the PI3K/Akt/mTOR pathway can support macrophage polarization toward an M2-like phenotype by promoting metabolic reprogramming processes such as oxidative phosphorylation and fatty acid oxidation (107, 109). Nascimento Júnior and colleagues provided important reverse genetic evidence, demonstrating that ketoconazole can effectively reverse M2 macrophage polarization by specifically disrupting the PI3K/Akt/mTOR pathway (110). However, the role of the PI3K/Akt/mTOR pathway in regulating macrophage polarization within the PAH-specific pathological environment still requires further experimental validation. These findings not only reveal the temporal dynamics of inflammatory responses during PAH progression but also provide an important basis for stage-specific targeted immunotherapy.

#### 5.3.4 PPAR-γ

PPAR-γ is a key transcription factor regulating macrophage M2 polarization. By promoting the expression of anti-inflammatory and tissue repair–related genes, it is central to the maintenance of immune homeostasis (111). Its activation involves binding of endogenous ligands (such as polyunsaturated fatty acids and prostaglandin derivatives) or synthetic agonists (such as thiazolidinediones) to the receptor, which promotes the formation of a heterodimer with the retinoid X receptor. This complex then recognizes and binds to PPAR response elements in the promoters of target genes, directly upregulating the expression of classical M2 markers including Arg1, CD206, and IL-10 (111, 112).On the other hand, PPAR-γ activation effectively antagonizes M1 polarization by inhibiting the activity of pro-inflammatory transcription factors such as NF-κB and AP-1. This inhibitory effect is mediated through multiple mechanisms, including direct protein–protein interactions, competitive recruitment of coactivators, and induction of anti-inflammatory gene expression (113). In PASMCs derived from idiopathic PAH patients, PPAR-γ is upregulated, indicating that its activation exerts protective effects on vascular cells in PAH (114). The PPAR-γ pathway’s influence on macrophages may represent an important therapeutic target in PAH.

### 5.4 Genetic and epigenetic modifications

Epigenetic mechanisms regulate gene expression without altering the DNA sequence and act as “molecular switches” in determining macrophage polarization fate. Such regulation occurs primarily at multiple levels, including DNA methylation, histone modifications, and non-coding RNAs (115).

#### 5.4.1 Methylation

DNA methylation, as a key epigenetic regulatory mechanism, involves the addition of methyl groups to cytosines in CpG dinucleotides by DNA methyltransferases. This modification can silence gene expression by blocking transcription factor binding or altering chromatin structure and is involved in the pathogenesis of numerous disorders (116). In PAH, studies using large-scale epigenome-wide association analyses have systematically revealed specific DNA methylation patterns in patients (117). Aberrant DNA methylation, together with histone modifications, is mechanistically linked to vascular remodeling and pulmonary vascular dysfunction in PAH (118).

Notably, DNA methylation is a key regulatory node in controlling macrophage polarization. During macrophage polarization, the expression of the DNA methyltransferase 3B (DNMT3B) is significantly reduced in M2 macrophages. Functional studies have shown that knockdown of DNMT3B promotes macrophage polarization toward the anti-inflammatory M2 phenotype and suppresses inflammation, whereas overexpression drives pro-inflammatory M1 polarization and exacerbates inflammatory responses. The molecular basis of this mechanism lies in DNMT3B’s direct regulation of the methylation status of the PPAR-γ promoter (119). Additionally, studies have found that DNMT3A expression is decreased in peripheral blood mononuclear cells from PAH patients. Hematopoietic-specific deletion of Dnmt3a in mice leads to spontaneous development of a PAH phenotype, which is further exacerbated under hypoxic conditions. This model exhibits increased pulmonary macrophages and elevated plasma IL-13 levels, while IL-1β antibody treatment effectively alleviates disease progression. These findings indicate that DNMT3A deficiency drives PAH development by promoting inflammatory responses (120).

N6-methyladenosine (m6A) methylation, the dominant internal modification of eukaryotic mRNA, dynamically regulates mRNA transcription, splicing, translation, and degradation through the coordinated actions of methyltransferases, demethylases, and binding proteins. Recent studies have revealed that m6A RNA methylation contributes to the pathogenesis of PAH by modulating macrophage polarization (121). At the molecular level, The m6A methyltransferase methyltransferase-like 3 is significantly upregulated in the lungs and macrophages of PAH models. Loss of methyltransferase-like 3 effectively regulates macrophage polarization by suppressing CD86^+^ M1 macrophages and associated pro-inflammatory cytokine expression, while promoting CD206^+^ M2 macrophages and anti-inflammatory cytokine production, along with downregulating fibrosis-related genes (122). Additionally, the m6A reader protein YTHDF2 is upregulated in AMs during early hypoxia, where it mediates m6A-dependent degradation of heme oxygenase 1 mRNA, thereby suppressing heme oxygenase 1 expression and influencing macrophage polarization and oxidative stress processes (123).

#### 5.4.2 Histone modifications

Histone modifications, as key epigenetic regulatory mechanisms, play a central role in macrophage polarization by dynamically modulating chromatin accessibility and transcription factor binding. Current studies indicate that histone acetylation and deacetylation, mediated by histone acetyltransferases and histone deacetylases (HDACs), can rapidly regulate the transcriptional activity of inflammation-related genes. In contrast, histone methylation, such as H3K9me2 and H3K27me3, catalyzed by methyltransferases like G9a and EZH2, contributes to the maintenance of more stable phenotypic states (124). Notably, the functions of these modifying enzymes are highly context-dependent: for example, G9a can enhance pro-inflammatory gene expression through promoting H3K9me2 modification, driving M1 polarization (125); whereas in specific microenvironments, EZH2 can regulate the expression of M2-related genes by modulating signaling pathways such as STAT6 and PI3K/Akt (126).

In the pathological environment of PAH, aberrant expression of histone-modifying enzymes is closely associated with disease progression. Studies have shown that a group of HDACs, including HDAC1, HDAC2, and HDAC3, are upregulated in pulmonary vascular cells from PAH patients and animal models, and that HDAC inhibitors can effectively suppress pro-inflammatory mediator production, reduce cell proliferation, and ameliorate vascular remodeling (127). Similarly, the histone methyltransferase G9a and its complexes are functionally upregulated in PAH models, and their inhibition via the selective inhibitor BIX01294 or genetic interventions significantly improves pulmonary vascular lesions and RV function (128). Additionally, EZH2 is highly expressed in hypoxia-induced PAH models, contributing to disease progression by promoting pulmonary artery smooth muscle cell proliferation and anti-apoptotic phenotypic switching (129).

Mechanistically, histone modifications regulate macrophage polarization in PAH through both indirect and direct pathways: the indirect pathway involves vascular wall cells, including endothelial cells, smooth muscle cells, and fibroblasts, which secrete chemokines and inflammatory mediators via histone modification–dependent mechanisms, thereby creating a microenvironment favorable for the polarization of specific macrophage phenotypes; the direct pathway involves histone-modifying enzymes within macrophages themselves, which alter the chromatin state of key transcription factors and metabolic genes to determine their polarization direction (118, 127, 128).

#### 5.4.3 Non-coding RNA

In recent years, non-coding RNAs (ncRNAs) have emerged as key players in the pathogenesis of PAH. (130). In the human genome, over 95% of transcripts are ncRNAs, with microRNAs (miRNAs) and long non-coding RNAs (lncRNAs) being the most extensively studied. miRNAs, a class of non-coding RNA molecules 19–25 nucleotides in length, regulate gene expression by mediating post-transcriptional silencing of target mRNAs (131). In contrast, lncRNAs, characterized by a length of over 200 nucleotides, exert multi-level regulatory functions through interactions with proteins, RNAs, or DNA (130).

In the pathological progression of PAH, aberrant activation of macrophages and inflammation in the lung parenchyma constitute central mechanisms. Evidence has demonstrated that mesenchymal stem cell–derived exosomes (MSC-Exos) can significantly inhibit macrophage infiltration into lung tissue and reduce the release of inflammatory cytokines such as CCL2 and IL-6 by suppressing STAT3 activity and modulating the expression of miRNAs including miR-34a and miR-124 (132). This regulatory mechanism is corroborated by findings in myocardial injury models: miR-182 promotes M2 polarization via the TLR4/NF-κB/PI3K signaling axis, thereby improving myocardial ischemia–reperfusion injury (133), whereas miR-155 promotes M1 polarization through TLR signaling, exacerbating myocardial inflammation (134), collectively highlighting a conserved role of miRNAs in regulating macrophage polarization.

With the advancement of research, multiple key miRNAs that regulate macrophage polarization in PAH have been identified. In PAH rat models, miR-200b is significantly downregulated in lung tissue and macrophages, and facilitates the delivery of miR-200b by bone marrow–derived mesenchymal stem cell extracellular vesicles, which promotes macrophage polarization toward the anti-inflammatory M2 phenotype. (135). Similarly, miR-29a-3p exerts anti-inflammatory effects in macrophages by targeting and suppressing ENPP2 expression (136). Conversely, exosomes derived from M1 macrophages can promote disease progression by delivering miR-663b, which enhances pulmonary artery smooth muscle cell proliferation, migration, inflammation, and oxidative stress by inhibiting the AMPK/Sirt1 pathway, thereby attenuating its protective signaling. (137). Collectively, these findings reveal that miRNAs from different sources and with distinct functions finely regulate macrophage polarization, forming a complex molecular regulatory network in the pathogenesis of PAH.

In addition to miRNAs, lncRNAs also play important roles in regulating macrophages in PAH. Studies have shown that multiple lncRNAs are abnormally expressed in PAH models and participate in pulmonary vascular pathology by modulating cell proliferation and migration (138). Notably, overexpression of specific lncRNAs can promote pulmonary artery smooth muscle cell proliferation, whereas their knockdown induces apoptosis, highlighting their critical role in pulmonary vascular remodeling (139). Importantly, the team led by Hou, using high-throughput sequencing, first demonstrated that the lncRNA NONRATT009275.2 can directly promote macrophage polarization toward the M2 phenotype (140). This groundbreaking finding not only elucidates a novel mechanism by which lncRNAs contribute to PAH pathogenesis through immune regulation but also provides a new direction for future research.

Chronic hypoxia, metabolic reprogramming, and epigenetic modifications together form a mutually reinforcing regulatory loop that locks macrophages into a pathogenic state. Chronic hypoxia stabilizes hypoxia-inducible factors, initiating metabolic reprogramming processes such as glycolysis, the pentose phosphate pathway, and fatty acid oxidation (82). The accumulated metabolites, including succinate and lactate, not only serve as metabolic precursors but also act as signaling molecules that drive epigenetic landscape alterations, including histone lactylation, acetylation, and m6A RNA methylation (92, 122). These epigenetic modifications continuously enhance the expression of pro-inflammatory and pro-fibrotic genes, ultimately consolidating macrophages into a stable pathogenic phenotype, promoting pulmonary vascular inflammation, and facilitating irreversible vascular remodeling. This “hypoxia → HIF → metabolic reprogramming → metabolites → epigenetic modifications → stable pathogenic phenotype” axis provides a theoretical basis for developing innovative therapeutic strategies aimed at disrupting this vicious cycle.

## 6 Therapeutic strategy

### 6.1 Recruitment/survival

#### 6.1.1 Targeting CSF1R signaling

This strategy aims to restore immune microenvironmental balance by targeting macrophage survival and polarization. The theoretical basis is that macrophage polarization imbalance alone is sufficient to induce PAH (44). In the Su/Hy rat model, the CSF1R inhibitor pexidartinib effectively reduced RV systolic pressure and the number of perivascular macrophages by inhibiting CSF1R and JNK signaling in M2 macrophages (141), demonstrating the therapeutic potential of this approach.

#### 6.1.2 Blocking the chemokine axis CCR2/CCR5

This strategy functions by blocking key signaling pathways that guide macrophage migration. CCR2 gene deletion markedly inhibits CCL2-induced PASMC hyperproliferation and reduces perivascular macrophage infiltration (142). Importantly, a dual CCR2/CCR5 antagonism strategy can effectively disrupt the pathological positive feedback loop between macrophages and PASMCs, exhibiting superior therapeutic efficacy compared with single-target inhibition (63).

#### 6.1.3 Intervening in developmental and inflammatory signaling

This strategy aims to target upstream signals that regulate macrophage activation. On one hand, direct intervention in developmental pathways, such as using the RUNX1 inhibitor Ro5-3335, can effectively block the recruitment and activation of pulmonary macrophages by inhibiting endothelial-to-hematopoietic cell transition (143). On the other hand, mechanistic studies provide new targets for intervention: loss of BMPR2 signaling in macrophages drives upregulation of pro-inflammatory factors (e.g., CXCL12, C5a) and vascular muscularization (144), while macrophage-derived PDGF-B is a key driver of disease progression (58). These findings collectively suggest that restoring BMPR2 function or blocking PDGF-B signaling represents a highly promising therapeutic approach.

### 6.2 Immunometabolism

#### 6.2.1 Glycolysis

Aberrant activation of glycolysis is a key driver of the pro-inflammatory phenotype in macrophages. Studies in MCT-induced PAH rat models have shown that β-catenin promotes lactate production and inflammatory cytokine expression by upregulating the activity of key glycolytic enzymes, thereby enhancing PASMC proliferation and migration; treatment with its inhibitor XAV939 effectively reduces glycolysis, suppresses inflammation, and alleviates pulmonary vascular remodeling and RV hypertrophy (145). Additionally, in multiple PAH models and patient samples, the glycolytic key enzyme PFKFB3 mediates macrophage inflammatory responses via regulation of the HIF-1a/HIF-2a signaling pathway, and its inhibitor 3PO reduces pulmonary macrophage numbers and pro-inflammatory cytokine secretion, effectively ameliorating PAH symptoms (82).

#### 6.2.2 Pentose phosphate pathway

In hypoxia-induced PAH models, targeting the glycolytic bypass enzyme G6PD pharmacologically decreases M2a macrophage markers, TNFα+ platelets, and pro-inflammatory factor accumulation in the lungs, suggesting its potential as a therapeutic target (146).

#### 6.2.3 Fatty acid oxidation

The fatty acid oxidation metabolic pathway is significantly activated in PAH. In multiple PAH models, including Sugen-hypoxia rats, schistosomiasis-induced, and hypoxia-induced mice, treatment with the fatty acid oxidation inhibitor etomoxir effectively improves pulmonary hemodynamics, promotes vascular de-remodeling, and attenuates endothelin-1–induced vasoconstriction. Its therapeutic effect is closely associated with a reduction in perivascular macrophage infiltration in the diseased lungs (147).

#### 6.2.4 Glutamine metabolism

Glutamine metabolism plays a critical role in macrophage inflammatory polarization. Studies in MCT-induced PAH rat models have shown that the glutaminase 1 inhibitor BPTES can suppress M1 macrophage polarization, NLRP3 activation, and the release of pro-inflammatory cytokines, thereby reducing PASMC proliferation and migration induced by inflammatory stimuli, ultimately ameliorating key pathological features of PAH in rats, including elevated pulmonary arterial pressure, impaired RV function, and vascular remodeling. (148).

#### 6.2.5 Other metabolism-related regulations

In addition to the aforementioned core metabolic pathways, other metabolism-related targets also contribute to the regulation of macrophage function. In hypoxia-induced PAH models, genetic deletion of serum- and glucocorticoid-regulated kinase 1 suppresses the expression of pro-inflammatory factors in macrophages and reduces macrophage infiltration in the lungs, thereby effectively inhibiting PAH progression (149). Studies in the SU/Hx rat model have shown that the carbonic anhydrase inhibitor acetazolamide exerts anti-inflammatory and anti-vascular remodeling effects by directly inhibiting macrophage carbonic anhydrase activity and inducing systemic metabolic acidosis, thereby cooperatively modulating M1/M2 macrophage polarization (150).

#### 6.2.6 HIF-1α

In hypoxic PAH mice and *in vitro* co-culture experiments, the metabolic sensor CtBP1 has been identified as a key regulator of fibroblast-induced transcriptional and metabolic reprogramming in macrophages; inhibition with MTOB suppresses its aberrant activation, alleviating perivascular inflammation and remodeling (151). In addition, myeloid-specific HIF-1α knockout mice exposed to chronic hypoxia exhibited a significantly attenuated PAH phenotype and reduced macrophage infiltration, due to decreased macrophage chemotactic capacity and lower ATP levels (74).

### 6.3 Epigenetic and non-coding RNA targets

Studies have shown that targeting epigenetic and signaling pathways is an effective strategy to modulate macrophage function in PAH. At the epigenetic level, inhibition of methyltransferase-like 3 (e.g., using STM2457) can regulate macrophage polarization via modulation of m6A methylation, thereby improving hemodynamics, attenuating pulmonary vascular remodeling and RV hypertrophy, and suppressing fibrosis (122). Similarly, HDAC inhibitors such as butyrate increase histone H3 acetylation, reduce macrophage accumulation and inflammatory cytokine expression, and prevent or reverse PAH pathological changes (152).Regulation of key signaling pathways is also critical: for example, macrophage-specific deletion of the crucial RNA enzyme Zinc Finger CCCH-Type Containing 12A (ZC3H12A/Regnase-1) aberrantly activates IL-6 and PDGF signaling, leading to spontaneous severe PAH in mice. (153). Targeting innate immune signaling, such as with stimulator of interferon genes inhibitor C-176 (154), TLR9 inhibitor E6446, or chloroquine (155), blocks NLRP3 inflammasome assembly and the subsequent NF-κB/IL-6 signaling cascade, significantly reducing macrophage-driven inflammation, cellular infiltration, vascular remodeling, and improving survival. In addition, selective NLRP3 inhibition with MCC950 has been shown to decrease M1 macrophage accumulation and improve RV function (156).

Targeting specific inflammatory mediators and proteases also shows therapeutic potential. Antagonists of macrophage migration inhibitory factor (MIF), such as MIF098 and ISO-1, inhibit pulmonary artery smooth muscle cell proliferation and collagen deposition by modulating MAPK/ERK and TGF-β1/Smad signaling, improving cardiopulmonary pathology in systemic lupus erythematosus– and idiopathic pulmonary fibrosis–associated PAH models (157, 158). Melatonin exerts dual effects via its membrane receptor, both reducing macrophage infiltration in the lung and directly regulating intracellular calcium homeostasis to suppress NLRP3 inflammasome activation, thereby alleviating vascular inflammation and leakage (159).

Furthermore, macrophage-specific deletion of the protease Legumain blocks MMP-2 activation and TGF-β1 maturation, effectively attenuating vascular remodeling (160). The bradykinin B1 receptor antagonist BI113823 reduces macrophage infiltration, downregulates iNOS, MMP-2/9, and ERK/AKT phosphorylation, reverses pulmonary neointima formation and fibrosis, and markedly improves right heart function (161).

### 6.4 Delivery platforms

Emerging delivery platforms and advanced therapeutic strategies have opened new avenues for disease-modifying treatment of PAH. The core advantage of these approaches lies in their ability to go beyond traditional single-target interventions, achieving more effective control over disease progression by precisely modulating macrophage functions while simultaneously influencing multiple pathological processes. From engineered extracellular vesicles and targeted nanoparticles to multi-target receptor fusion proteins and optimized natural products, these innovative strategies demonstrate significant advantages in drug delivery efficiency, immunomodulatory precision, and breadth of signaling pathway intervention.

The following table systematically summarizes the mechanisms of action and existing evidence for these approaches (**Table 1**):

**Table 1:** Macrophage polarization and functional specialization.

Intervention type	Intervention example	Model	Key findings & mechanisms	Clinical stage	References
Extracellular Vesicle Therapy	Tadalafil-preconditioned MSC Exosomes	Hypoxia-induced rat PAH model	Upregulates miR-29a-3p, exerts anti-inflammatory effects via macrophage modulation, inhibits SMC proliferation and migration	Preclinical	(136)
Extracellular Vesicle Therapy	Human Umbilical Cord MSC-derived Exosomes	Hypoxia-treated mice	Promotes macrophage polarization to M2 phenotype, inhibits IL-33/ST2 signaling axis	Preclinical	(162)
Genetically Modified Cell Therapy	7ND-gene Modified MSCs	BPD rat model	Significantly reduces M1 macrophage infiltration and pro-inflammatory cytokine expression	Preclinical	(163)
Nanoparticle Gene Therapy	Pdgfb siRNA-loaded Nanoparticles	Hypoxic mouse model	Targets lung macrophages, inhibits PDGF-B expression, blocks macrophage-SMC signaling axis	Preclinical	(58)
Receptor Fusion Protein	Sotatercept (ActRIIA-Fc)	Sugen-hypoxia rats & Bmpr2 haploinsufficient mice	Reverses macrophage-related pro-inflammatory gene profile	Clinical (Phase II/III)	(164)
Natural Product	Corosolic Acid (CRA)	MCT-PAH rat model	Regulates macrophage-driven inflammation by inhibiting PDGF receptor signaling axis	Preclinical	(165)
Natural Product	Celastrol	MCT-induced PAH rat model	Modulates macrophage-related inflammation via NF-κB signaling pathway	Preclinical	(166)
Natural Product/Hormone	Niacin	Su/Hx & MCT-induced PAH models	Activates H-PGDS in macrophages, increases PGD_2_ production	Preclinical	(167)
Natural Product/Drug	Donepezil	MCT-induced PAH rat model	Reduces macrophage infiltration and inhibits M2 macrophage activation	Preclinical	(168)
Natural Product/Hormone	Melatonin	PAH patients & animal models	Reduces macrophage infiltration and inhibits NLRP3 inflammasome activation	Preclinical	(159)
Natural Product/Extract	EPA-L	MCT-induced PAH rat model	Limited improvement on macrophage infiltration; primarily acts through other mechanisms	Preclinical	(169)

## 7 Challenges

Current research faces several major challenges, including the incomplete understanding of the complex regulatory networks underlying macrophage heterogeneity, pathological differences between animal models and human disease, and the lack of precise delivery systems targeting specific macrophage subsets. Future studies should focus on leveraging single-cell multi-omics technologies to comprehensively characterize the dynamic changes of macrophages within the pulmonary vascular microenvironment, developing cell-specific delivery platforms for precise intervention, and exploring individualized therapeutic strategies based on macrophage phenotyping. Additionally, establishing more reliable efficacy assessment systems and multicenter clinical translation platforms is essential to accelerate the transition of macrophage-targeted therapies from basic research to clinical application.

## 8 Conclusion and perspectives

Current evidence indicates that macrophages are central regulators of cross-organ inflammation and vascular remodeling in PAH. They act as pivotal mediators within the lung–heart axis, linking local pulmonary vascular inflammation to RV remodeling. Distinct macrophage subsets perform specific functions in amplifying inflammation, driving fibrosis, and mediating metabolism-related injury. Targeting macrophages shows significant therapeutic potential, but precise identification and modulation of pathogenic subsets remain critical for achieving effective intervention. Future research should focus on mapping macrophage subsets across species; selectively modulating pathogenic subsets without inducing immunosuppression; and evaluating potential synergistic effects with existing therapies.

## References

[1] Saleh KM Mallat J Mohammed S Bodi G Alazazzi H Salim S . Comparative efficacy and safety of prostacyclin therapies for pulmonary arterial hypertension: a systematic review and network meta-analysis. Front Med. (2025) 12:1643220. doi: 10.3389/fmed.2025.1643220 41158465 PMC12554579

[2] Prins KW Thenappan T . World health organization group I pulmonary hypertension: epidemiology and pathophysiology. Cardiol Clin. (2016) 34:363–74. doi: 10.1016/j.ccl.2016.04.001 27443134 PMC4959804

[3] Klouda T Yuan K . Inflammation in pulmonary arterial hypertension. Adv Exp Med Biol. (2021) 1303:351–72. doi: 10.1007/978-3-030-63046-1_19 33788202

[4] Ghofrani H-A Gomberg-Maitland M Zhao L Grimminger F . Mechanisms and treatment of pulmonary arterial hypertension. Nat Rev Cardiol. (2025) 22:105–20. doi: 10.1038/s41569-024-01064-4 39112561

[5] Crosswhite P Sun Z . Molecular mechanisms of pulmonary arterial remodeling. Mol Med Camb. Mass. (2014) 20:191–201. doi: 10.2119/molmed.2013.00165 24676136 PMC4002851

[6] Zuo Y Li B Gao M Xiong R He R Li N . Novel insights and new therapeutic potentials for macrophages in pulmonary hypertension. Respir Res. (2024) 25:147. doi: 10.1186/s12931-024-02772-8 38555425 PMC10981837

[7] Luo M Zhao F Cheng H Su M Wang Y . Macrophage polarization: an important role in inflammatory diseases. Front Immunol. (2024) 15:1352946. doi: 10.3389/fimmu.2024.1352946 38660308 PMC11039887

[8] Zhang M-Q Wang C-C Pang X-B Shi J-Z Li H-R Xie X-M . Role of macrophages in pulmonary arterial hypertension. Front Immunol. (2023) 14:1152881. doi: 10.3389/fimmu.2023.1152881 37153557 PMC10154553

[9] Jefferson L Lima PDA Archer SL . Macrophage plasticity and glucose metabolism: the role of immunometabolism in pulmonary arterial hypertension. Clin Sci Lond Engl. (2025) 138:CS20257363. doi: 10.1042/CS20257363 41032703 PMC12794329

[10] Spiekerkoetter E . Macrophages: key conductors behind perivascular inflammation and vascular remodeling in hypoxia-induced pulmonary hypertension. J Clin Invest. (2025) 135:e190957. doi: 10.1172/JCI190957 40091832 PMC11910220

[11] van Furth R Cohn ZA . The origin and kinetics of mononuclear phagocytes. J Exp Med. (1968) 128:415–35. doi: 10.1084/jem.128.3.415 5666958 PMC2138527

[12] Hoeffel G Ginhoux F . Fetal monocytes and the origins of tissue-resident macrophages. Cell Immunol. (2018) 330:5–15. doi: 10.1016/j.cellimm.2018.01.001 29475558

[13] Shi C Pamer EG . Monocyte recruitment during infection and inflammation. Nat Rev Immunol. (2011) 11:762–74. doi: 10.1038/nri3070 21984070 PMC3947780

[14] Zhao Y Zou W Du J Zhao Y . The origins and homeostasis of monocytes and tissue-resident macrophages in physiological situation. J Cell Physiol. (2018) 233:6425–39. doi: 10.1002/jcp.26461 29323706

[15] Tan SYS Krasnow MA . Developmental origin of lung macrophage diversity. Dev Camb. Engl. (2016) 143:1318–27. doi: 10.1242/dev.129122 26952982 PMC4852511

[16] Harper RL Zhou X Marciano DP Cao A Wang L Chen G . Altered maturation and activation state of circulating monocytes is associated with their enhanced recruitment in pulmonary arterial hypertension. Respir Res. (2025) 26:148. doi: 10.1186/s12931-025-03182-0 40234964 PMC11998417

[17] Florentin J Coppin E Vasamsetti SB Zhao J Tai Y-Y Tang Y . Inflammatory macrophage expansion in pulmonary hypertension depends upon mobilization of blood-borne monocytes. J Immunol Baltim. Md. (2018) 200:3612–25. doi: 10.4049/jimmunol.1701287 29632145 PMC5940510

[18] Willis GR Fernandez-Gonzalez A Reis M Mitsialis SA Kourembanas S . Macrophage immunomodulation: the gatekeeper for mesenchymal stem cell derived-exosomes in pulmonary arterial hypertension? Int J Mol Sci. (2018) 19:2534. doi: 10.3390/ijms19092534 30150544 PMC6164282

[19] Yan L Wang J Cai X Liou Y-C Shen H-M Hao J . Macrophage plasticity: signaling pathways, tissue repair, and regeneration. MedComm. (2024) 5:e658. doi: 10.1002/mco2.658 39092292 PMC11292402

[20] Shapouri-Moghaddam A Mohammadian S Vazini H Taghadosi M Esmaeili S-A Mardani F . Macrophage plasticity, polarization, and function in health and disease. J Cell Physiol. (2018) 233:6425–40. doi: 10.1002/jcp.26429 29319160 PMC13482560

[21] Crayne CB Albeituni S Nichols KE Cron RQ . The immunology of macrophage activation syndrome. Front Immunol. (2019) 10:119. doi: 10.3389/fimmu.2019.00119 30774631 PMC6367262

[22] Ni D Zhou H Wang P Xu F Li C . Visualizing macrophage phenotypes and polarization in diseases: from biomarkers to molecular probes. Phenomics Cham Switz. (2023) 3:613–38. doi: 10.1007/s43657-023-00129-7 38223685 PMC10781933

[23] Yunna C Mengru H Lei W Weidong C . Macrophage M1/M2 polarization. Eur J Pharmacol. (2020) 877:173090. doi: 10.1016/j.ejphar.2020.173090 32234529

[24] Rőszer T . Understanding the mysterious M2 macrophage through activation markers and effector mechanisms. Mediators Inflamm. (2015) 2015:816460. doi: 10.1155/2015/816460 26089604 PMC4452191

[25] Wynn TA Vannella KM . Macrophages in tissue repair, regeneration, and fibrosis. Immunity. (2016) 44:450–62. doi: 10.1016/j.immuni.2016.02.015 26982353 PMC4794754

[26] Liu L Stokes JV Tan W Pruett SB . An optimized flow cytometry panel for classifying macrophage polarization. J Immunol Methods. (2022) 511:113378. doi: 10.1016/j.jim.2022.113378 36265578

[27] Gharavi AT Hanjani NA Movahed E Doroudian M . The role of macrophage subtypes and exosomes in immunomodulation. Cell Mol Biol Lett. (2022) 27:83. doi: 10.1186/s11658-022-00384-y 36192691 PMC9528143

[28] Jiang Y Cai R Huang Y Zhu L Xiao L Wang C . Macrophages in organ fibrosis: from pathogenesis to therapeutic targets. Cell Death Discov. (2024) 10:487. doi: 10.1038/s41420-024-02247-1 39632841 PMC11618518

[29] Tian L Yu Q Liu D Chen Z Zhang Y Lu J . Epithelial-mesenchymal transition of peritoneal mesothelial cells is enhanced by M2c macrophage polarization. Immunol Invest. (2022) 51:301–15. doi: 10.1080/08820139.2020.1828911 34490837

[30] Wang Q Ni H Lan L Wei X Xiang R Wang Y . Fra-1 protooncogene regulates IL-6 expression in macrophages and promotes the generation of M2d macrophages. Cell Res. (2010) 20:701–12. doi: 10.1038/cr.2010.52 20386569

[31] Jiang P Huang H Xie M Liu Z Jiang L Shi H . Single-cell characterization of the immune heterogeneity of pulmonary hypertension identifies novel targets for immunotherapy. BMC Immunol. (2025) 26:5. doi: 10.1186/s12865-025-00684-w 39930365 PMC11809027

[32] Tang B Vadgama A Redmann B Hong J . Charting the cellular landscape of pulmonary arterial hypertension through single-cell omics. Respir Res. (2024) 25:192. doi: 10.1186/s12931-024-02823-0 38702687 PMC11067161

[33] Mao X Li Y Yang R Wei J Zhao Z Zhang T . Single-cell RNA-sequencing reveals the active involvement of macrophage polarizations in pulmonary hypertension. Dis Markers. (2022) 2022:5398157. doi: 10.1155/2022/5398157 36246557 PMC9553540

[34] Rabinovitch M Guignabert C Humbert M Nicolls MR . Inflammation and immunity in the pathogenesis of pulmonary arterial hypertension. Circ Res. (2014) 115:165–75. doi: 10.1161/CIRCRESAHA.113.301141 24951765 PMC4097142

[35] Fan Y Hao Y Gao D Li G Zhang Z . Phenotype and function of macrophage polarization in monocrotaline-induced pulmonary arterial hypertension rat model. Physiol Res. (2021) 70:213–26. doi: 10.33549/physiolres.934456 33676385 PMC8820576

[36] Ichikawa A Kuba K Morita M Chida S Tezuka H Hara H . CXCL10-CXCR3 enhances the development of neutrophil-mediated fulminant lung injury of viral and nonviral origin. Am J Respir Crit Care Med. (2013) 187:65–77. doi: 10.1164/rccm.201203-0508OC 23144331 PMC3927876

[37] Amsellem V Abid S Poupel L Parpaleix A Rodero M Gary-Bobo G . Roles for the CX3CL1/CX3CR1 and CCL2/CCR2 chemokine systems in hypoxic pulmonary hypertension. Am J Respir Cell Mol Biol. (2017) 56:597–608. doi: 10.1165/rcmb.2016-0201OC 28125278

[38] Liu H Wang Y Zhang Q Liu C Ma Y Huang P . Macrophage-derived inflammation promotes pulmonary vascular remodeling in hypoxia-induced pulmonary arterial hypertension mice. Immunol Lett. (2023) 263:113–22. doi: 10.1016/j.imlet.2023.10.005 37875238

[39] Kumar R Nolan K Kassa B Chanana N Palmo T Sharma K . Monocytes and interstitial macrophages contribute to hypoxic pulmonary hypertension. J Clin Invest. (2025) 135:e176865. doi: 10.1172/JCI176865 39883518 PMC11910231

[40] Ross DJ Strieter RM Fishbein MC Ardehali A Belperio JA . Type I immune response cytokine-chemokine cascade is associated with pulmonary arterial hypertension. J Heart Lung Transplant. Off Publ. Int Soc Heart Transplant. (2012) 31:865–73. doi: 10.1016/j.healun.2012.04.008 22658713

[41] Mantovani A Sica A Locati M . Macrophage polarization comes of age. Immunity. (2005) 23:344–6. doi: 10.1016/j.immuni.2005.10.001 16226499

[42] Minton K . Immune regulation: IL-10 targets macrophage metabolism. Nat Rev Immunol. (2017) 17:345. doi: 10.1038/nri.2017.57 28548135

[43] Wang J Zhao X Wan YY . Intricacies of TGF-β signaling in Treg and Th17 cell biology. Cell Mol Immunol. (2023) 20:1002–22. doi: 10.1038/s41423-023-01036-7 37217798 PMC10468540

[44] Zawia A Arnold ND West L Pickworth JA Turton H Iremonger J . Altered macrophage polarization induces experimental pulmonary hypertension and is observed in patients with pulmonary arterial hypertension. Arterioscler Thromb Vasc Biol. (2021) 41:430–45. doi: 10.1161/ATVBAHA.120.314639 33147993 PMC7752239

[45] Al-Qazazi R Lima PDA Prisco SZ Potus F Dasgupta A Chen K-H . Macrophage-NLRP3 activation promotes right ventricle failure in pulmonary arterial hypertension. Am J Respir Crit Care Med. (2022) 206:608–24. doi: 10.1164/rccm.202110-2274OC 35699679 PMC9716901

[46] Fernandez-Gonzalez A Mukhia A Nadkarni J Willis GR Reis M Zhumka K . Immunoregulatory macrophages modify local pulmonary immunity and ameliorate hypoxic pulmonary hypertension. Arterioscler Thromb Vasc Biol. (2024) 44:e288–303. doi: 10.1161/ATVBAHA.124.321264 39387119 PMC11697987

[47] Kumar S Mickael C Kumar R Prasad RR Campbell NV Zhang H . Single cell transcriptomic analyses reveal diverse and dynamic changes of distinct populations of lung interstitial macrophages in hypoxia-induced pulmonary hypertension. Front Immunol. (2024) 15:1372959. doi: 10.3389/fimmu.2024.1372959 38690277 PMC11059952

[48] Cho JG Lee A Chang W Lee M-S Kim J . Endothelial to mesenchymal transition represents a key link in the interaction between inflammation and endothelial dysfunction. Front Immunol. (2018) 9:294. doi: 10.3389/fimmu.2018.00294 29515588 PMC5826197

[49] Good RB Gilbane AJ Trinder SL Denton CP Coghlan G Abraham DJ . Endothelial to mesenchymal transition contributes to endothelial dysfunction in pulmonary arterial hypertension. Am J Pathol. (2015) 185:1850–8. doi: 10.1016/j.ajpath.2015.03.019 25956031

[50] Chi P-L Cheng C-C Hung C-C Wang M-T Liu H-Y Ke M-W . MMP-10 from M1 macrophages promotes pulmonary vascular remodeling and pulmonary arterial hypertension. Int J Biol Sci. (2022) 18:331–48. doi: 10.7150/ijbs.66472 34975336 PMC8692144

[51] Rodor J Chen SH Scanlon JP Monteiro JP Caudrillier A Sweta S . Single-cell RNA sequencing profiling of mouse endothelial cells in response to pulmonary arterial hypertension. Cardiovasc Res. (2022) 118:2519–34. doi: 10.1093/cvr/cvab296 34528097 PMC9400412

[52] Yang H Cheng H Dai R Shang L Zhang X Wen H . Macrophage polarization in tissue fibrosis. PeerJ. (2023) 11:e16092. doi: 10.7717/peerj.16092 37849830 PMC10578305

[53] Ma W Ma Y Bai Y Su X . Changes in macrophages in pulmonary hypertension: A focus on high-altitude pulmonary hypertension. Anatol. J Cardiol. (2025) 29:210–21. doi: 10.14744/AnatolJCardiol.2025.5013 40062372 PMC12053306

[54] Yung L-M Yang P Joshi S Augur ZM Kim SSJ Bocobo GA . ACTRIIA-Fc rebalances activin/GDF versus BMP signaling in pulmonary hypertension. Sci Transl Med. (2020) 12:eaaz5660. doi: 10.1126/scitranslmed.aaz5660 32404506 PMC8259900

[55] Andre P Joshi SR Briscoe SD Alexander MJ Li G Kumar R . Therapeutic approaches for treating pulmonary arterial hypertension by correcting imbalanced TGF-β Superfamily signaling. Front Med. (2021) 8:814222. doi: 10.3389/fmed.2021.814222 35141256 PMC8818880

[56] Zaiman AL Podowski M Medicherla S Gordy K Xu F Zhen L . Role of the TGF-beta/Alk5 signaling pathway in monocrotaline-induced pulmonary hypertension. Am J Respir Crit Care Med. (2008) 177:896–905. doi: 10.1164/rccm.200707-1083OC 18202349 PMC2292828

[57] Calvier L Chouvarine P Legchenko E Kokeny G Mozes MM Hansmann G . Chronic TGF-β1 signaling in pulmonary arterial hypertension induces sustained canonical smad3 pathways in vascular smooth muscle cells. Am J Respir Cell Mol Biol. (2019) 61:121–3. doi: 10.1165/rcmb.2018-0275LE 31259625

[58] Ntokou A Dave JM Kauffman AC Sauler M Ryu C Hwa J . Macrophage-derived PDGF-B induces muscularization in murine and human pulmonary hypertension. JCI Insight. (2021) 6:139067. doi: 10.1172/jci.insight.139067 33591958 PMC8026182

[59] Yamamura A . Growth factors involved in vascular remodeling in pulmonary arterial hypertension. J Smooth Muscle Res Nihon Heikatsukin Gakkai Kikanshi. (2025) 61:82–92. doi: 10.1540/jsmr.61.82 40903242 PMC12418036

[60] Dudley AC Griffioen AW . Pathological angiogenesis: mechanisms and therapeutic strategies. Angiogenesis. (2023) 26:313–47. doi: 10.1007/s10456-023-09876-7 37060495 PMC10105163

[61] Wang R-R Yuan T-Y Wang J-M Chen Y-C Zhao J-L Li M-T . Immunity and inflammation in pulmonary arterial hypertension: From pathophysiology mechanisms to treatment perspective. Pharmacol Res. (2022) 180:106238. doi: 10.1016/j.phrs.2022.106238 35504356

[62] Boucherat O Agrawal V Lawrie A Bonnet S . The latest in animal models of pulmonary hypertension and right ventricular failure. Circ Res. (2022) 130:1466–86. doi: 10.1161/CIRCRESAHA.121.319971 35482834 PMC9060385

[63] Abid S Marcos E Parpaleix A Amsellem V Breau M Houssaini A . CCR2/CCR5-mediated macrophage-smooth muscle cell crosstalk in pulmonary hypertension. Eur Respir J. (2019) 54:1802308. doi: 10.1183/13993003.02308-2018 31320454

[64] Varzideh F Forzano I Farroni E Mone P Kansakar U Santulli G . Macrophages regulate inflammatory vascular remodeling in pulmonary hypertension. Hypertens Dallas Tex. (2025) 82:460–2. doi: 10.1161/HYPERTENSIONAHA.124.24309 39970256 PMC11841922

[65] Hemnes AR Celermajer DS D’Alto M Haddad F Hassoun PM Prins KW . Pathophysiology of the right ventricle and its pulmonary vascular interaction. Eur Respir J. (2024) 64:2401321. doi: 10.1183/13993003.01321-2024 39209482 PMC11525331

[66] Ryan JJ Archer SL . The right ventricle in pulmonary arterial hypertension: disorders of metabolism, angiogenesis and adrenergic signaling in right ventricular failure. Circ Res. (2014) 115:176–88. doi: 10.1161/CIRCRESAHA.113.301129 24951766 PMC4112290

[67] Rosenkranz S Howard LS Gomberg-Maitland M Hoeper MM . Systemic consequences of pulmonary hypertension and right-sided heart failure. Circulation. (2020) 141:678–93. doi: 10.1161/CIRCULATIONAHA.116.022362 32091921

[68] Bekedam FT Goumans MJ Bogaard HJ de Man FS Llucià-Valldeperas A . Molecular mechanisms and targets of right ventricular fibrosis in pulmonary hypertension. Pharmacol Ther. (2023) 244:108389. doi: 10.1016/j.pharmthera.2023.108389 36940790

[69] Gu S Mickael C Kumar R Lee MH Sanders L Kassa B . The role of macrophages in right ventricular remodeling in experimental pulmonary hypertension. Pulm. Circ. (2022) 12:e12105. doi: 10.1002/pul2.12105 35874852 PMC9297026

[70] Dewachter L Dewachter C . Inflammation in right ventricular failure: does it matter? Front Physiol. (2018) 9:1056. doi: 10.3389/fphys.2018.01056 30177883 PMC6109764

[71] Krock BL Skuli N Simon MC . Hypoxia-induced angiogenesis: good and evil. Genes Cancer. (2011) 2:1117–33. doi: 10.1177/1947601911423654 22866203 PMC3411127

[72] Semenza GL . Hypoxia-inducible factors in physiology and medicine. Cell. (2012) 148:399–408. doi: 10.1016/j.cell.2012.01.021 22304911 PMC3437543

[73] Pullamsetti SS Mamazhakypov A Weissmann N Seeger W Savai R . Hypoxia-inducible factor signaling in pulmonary hypertension. J Clin Invest. (2020) 130:5638–51. doi: 10.1172/JCI137558 32881714 PMC7598042

[74] Kojima H Tokunou T Takahara Y Sunagawa K Hirooka Y Ichiki T . Hypoxia-inducible factor-1 α deletion in myeloid lineage attenuates hypoxia-induced pulmonary hypertension. Physiol Rep. (2019) 7:e14025. doi: 10.14814/phy2.14025 30927327 PMC6440913

[75] El Kasmi KC Pugliese SC Riddle SR Poth JM Anderson AL Frid MG . Adventitial fibroblasts induce a distinct proinflammatory/profibrotic macrophage phenotype in pulmonary hypertension. J Immunol Baltim. Md. (2014) 193:597–609. doi: 10.4049/jimmunol.1303048 24928992 PMC4100597

[76] Imtiyaz HZ Williams EP Hickey MM Patel SA Durham AC Yuan L-J . Hypoxia-inducible factor 2alpha regulates macrophage function in mouse models of acute and tumor inflammation. J Clin Invest. (2010) 120:2699–714. doi: 10.1172/JCI39506 20644254 PMC2912179

[77] Labrousse-Arias D Castillo-González R Rogers NM Torres-Capelli M Barreira B Aragonés J . HIF-2α-mediated induction of pulmonary thrombospondin-1 contributes to hypoxia-driven vascular remodelling and vasoconstriction. Cardiovasc Res. (2016) 109:115–30. doi: 10.1093/cvr/cvv243 26503986 PMC4692290

[78] Tang H Babicheva A McDermott KM Gu Y Ayon RJ Song S . Endothelial HIF-2α contributes to severe pulmonary hypertension due to endothelial-to-mesenchymal transition. Am J Physiol Lung Cell Mol Physiol. (2018) 314:L256–75. doi: 10.1152/ajplung.00096.2017 29074488 PMC5866501

[79] Pokharel MD Marciano DP Fu P Franco MC Unwalla H Tieu K . Metabolic reprogramming, oxidative stress, and pulmonary hypertension. Redox Biol. (2023) 64:102797. doi: 10.1016/j.redox.2023.102797 37392518 PMC10363484

[80] Mills EL O’Neill LA . Reprogramming mitochondrial metabolism in macrophages as an anti-inflammatory signal. Eur J Immunol. (2016) 46:13–21. doi: 10.1002/eji.201445427 26643360

[81] Viola A Munari F Sánchez-Rodríguez R Scolaro T Castegna A . The metabolic signature of macrophage responses. Front Immunol. (2019) 10:1462. doi: 10.3389/fimmu.2019.01462 31333642 PMC6618143

[82] Wang L Zhang X Cao Y Ma Q Mao X Xu J . Mice with a specific deficiency of Pfkfb3 in myeloid cells are protected from hypoxia-induced pulmonary hypertension. Br J Pharmacol. (2021) 178:1055–72. doi: 10.1111/bph.15339 33300142

[83] Brittain EL Talati M Fessel JP Zhu H Penner N Calcutt MW . Fatty acid metabolic defects and right ventricular lipotoxicity in human pulmonary arterial hypertension. Circulation. (2016) 133:1936–44. doi: 10.1161/CIRCULATIONAHA.115.019351 27006481 PMC4870107

[84] Piao L Fang Y-H Parikh K Ryan JJ Toth PT Archer SL . Cardiac glutaminolysis: a maladaptive cancer metabolism pathway in the right ventricle in pulmonary hypertension. J Mol Med Berl. Ger. (2013) 91:1185–97. doi: 10.1007/s00109-013-1064-7 23794090 PMC3783571

[85] Van den Bossche J Baardman J Otto NA van der Velden S Neele AE van den Berg SM . Mitochondrial dysfunction prevents repolarization of inflammatory macrophages. Cell Rep. (2016) 17:684–96. doi: 10.1016/j.celrep.2016.09.008 27732846

[86] Rath M Müller I Kropf P Closs EI Munder M . Metabolism via arginase or nitric oxide synthase: two competing arginine pathways in macrophages. Front Immunol. (2014) 5:532. doi: 10.3389/fimmu.2014.00532 25386178 PMC4209874

[87] Kao CC Wedes SH Hsu JW Bohren KM Comhair SAA Jahoor F . Arginine metabolic endotypes in pulmonary arterial hypertension. Pulm. Circ. (2015) 5:124–34. doi: 10.1086/679720 25992277 PMC4405713

[88] Infantino V Iacobazzi V Palmieri F Menga A . ATP-citrate lyase is essential for macrophage inflammatory response. Biochem Biophys Res Commun. (2013) 440:105–11. doi: 10.1016/j.bbrc.2013.09.037 24051091

[89] Lauterbach MA Hanke JE Serefidou M Mangan MSJ Kolbe C-C Hess T . Toll-like receptor signaling rewires macrophage metabolism and promotes histone acetylation via ATP-citrate lyase. Immunity. (2019) 51:997–1011.e7. doi: 10.1016/j.immuni.2019.11.009 31851905

[90] Grobs Y Romanet C Lemay S-E Bourgeois A Voisine P Theberge C . ATP citrate lyase drives vascular remodeling in systemic and pulmonary vascular diseases through metabolic and epigenetic changes. Sci Transl Med. (2024) 16:eado7824. doi: 10.1126/scitranslmed.ado7824 39661707

[91] Chen J Zhang M Liu Y Zhao S Wang Y Wang M . Histone lactylation driven by mROS-mediated glycolytic shift promotes hypoxic pulmonary hypertension. J Mol Cell Biol. (2023) 14:mjac073. doi: 10.1093/jmcb/mjac073 36564027 PMC10175659

[92] Fan H Yang F Xiao Z Luo H Chen H Chen Z . Lactylation: novel epigenetic regulatory and therapeutic opportunities. Am J Physiol Endocrinol Metab. (2023) 324:E330–8. doi: 10.1152/ajpendo.00159.2022 36856188

[93] Farkas D Alhussaini AA Kraskauskas D Kraskauskiene V Cool CD Nicolls MR . Nuclear factor κB inhibition reduces lung vascular lumen obliteration in severe pulmonary hypertension in rats. Am J Respir Cell Mol Biol. (2014) 51:413–25. doi: 10.1165/rcmb.2013-0355OC 24684441 PMC4189489

[94] Kimura S Egashira K Chen L Nakano K Iwata E Miyagawa M . Nanoparticle-mediated delivery of nuclear factor kappaB decoy into lungs ameliorates monocrotaline-induced pulmonary arterial hypertension. Hypertens Dallas Tex. (2009) 53:877–83. doi: 10.1161/HYPERTENSIONAHA.108.121418 19307469

[95] Hong J Arneson D Umar S Ruffenach G Cunningham CM Ahn IS . Single-cell study of two rat models of pulmonary arterial hypertension reveals connections to human pathobiology and drug repositioning. Am J Respir Crit Care Med. (2021) 203:1006–22. doi: 10.1164/rccm.202006-2169OC 33021809 PMC8048757

[96] Ye Y Wang Y Yang Y Tao L . Aloperine suppresses LPS-induced macrophage activation through inhibiting the TLR4/NF-κB pathway. Inflamm Res Off J Eur Histamine Res Soc Al. (2020) 69:375–83. doi: 10.1007/s00011-019-01313-0 32144444

[97] Owen AM Luan L Burelbach KR McBride MA Stothers CL Boykin OA . MyD88-dependent signaling drives toll-like receptor-induced trained immunity in macrophages. Front Immunol. (2022) 13:1044662. doi: 10.3389/fimmu.2022.1044662 36439136 PMC9692127

[98] Liu B Wang X Chen T-Z Li G-L Tan C-C Chen Y . Polarization of M1 tumor associated macrophage promoted by the activation of TLR3 signal pathway. Asian Pac. J Trop Med. (2016) 9:484–8. doi: 10.1016/j.apjtm.2016.03.019 27261859

[99] Wang L Wang J Han L Chen T . Palmatine attenuated lipopolysaccharide-induced acute lung injury by inhibiting M1 phenotype macrophage polarization via NAMPT/TLR2/CCR1 signaling. J Agric Food Chem. (2024), 8871–9522. doi: 10.1021/acs.jafc.3c05597 38619332

[100] Yang S-R Yao H Rajendrasozhan S Chung S Edirisinghe I Valvo S . RelB is differentially regulated by IkappaB Kinase-alpha in B cells and mouse lung by cigarette smoke. Am J Respir Cell Mol Biol. (2009) 40:147–58. doi: 10.1165/rcmb.2008-0207OC 18688039 PMC2633139

[101] Chen T Su S Yang Z Zhang D Li Z Lu D . Srolo Bzhtang reduces inflammation and vascular remodeling via suppression of the MAPK/NF-κB signaling pathway in rats with pulmonary arterial hypertension. J Ethnopharmacol. (2022) 297:115572. doi: 10.1016/j.jep.2022.115572 35872290

[102] Zuo ZT Ma Y Sun Y Bai CQ Zhou HY Chen BH . Role of TLR4/NF-κB signalling pathway in pulmonary arterial hypertension in patients with chronic obstructive pulmonary disease. J Coll Physicians Surg.–Pak. JCPSP. (2020) 30:568–73. doi: 10.29271/jcpsp.2020.06.568 32703338

[103] Xia T Zhang M Lei W Yang R Fu S Fan Z . Advances in the role of STAT3 in macrophage polarization. Front Immunol. (2023) 14:1160719. doi: 10.3389/fimmu.2023.1160719 37081874 PMC10110879

[104] Liu Y Liu Z Tang H Shen Y Gong Z Xie N . The N6-methyladenosine (m6A)-forming enzyme METTL3 facilitates M1 macrophage polarization through the methylation of STAT1 mRNA. Am J Physiol Cell Physiol. (2019) 317:C762–75. doi: 10.1152/ajpcell.00212.2019 31365297

[105] Tang C Luo Y Li S Huang B Xu S Li L . Characteristics of inflammation process in monocrotaline-induced pulmonary arterial hypertension in rats. Biomed Pharmacother. Biomedecine Pharmacother. (2021) 133:111081. doi: 10.1016/j.biopha.2020.111081 33378977

[106] He B Shao B Cheng C Ye Z Yang Y Fan B . miR-21-mediated endothelial senescence and dysfunction are involved in cigarette smoke-induced pulmonary hypertension through activation of PI3K/AKT/mTOR signaling. Toxics. (2024) 12:396. doi: 10.3390/toxics12060396 38922076 PMC11209295

[107] Fedor A Bryniarski K Nazimek K . mTOR signaling in macrophages: all depends on the context. Int J Mol Sci. (2025) 26:7598. doi: 10.3390/ijms26157598 40806725 PMC12347599

[108] Zhang T Jiang X Diana C Zhao X Huang J Zhang Y . YTHDF1-mediated m6A modification of TOP2A drives pulmonary hypertension via the PI3K/Akt/mTOR pathway. Cell Signal. (2025) 134:111917. doi: 10.1016/j.cellsig.2025.111917 40451562

[109] Kadomoto S Izumi K Mizokami A . Macrophage polarity and disease control. Int J Mol Sci. (2021) 23:144. doi: 10.3390/ijms23010144 35008577 PMC8745226

[110] Nascimento Júnior JX Sola-Penna M Zancan P . Clotrimazole reverses macrophage M2 polarization by disrupting the PI3K/AKT/mTOR pathway. Biochem Biophys Res Commun. (2024) 696:149455. doi: 10.1016/j.bbrc.2023.149455 38176247

[111] Tu Y Liu J Kong D Guo X Li J Long Z . Irisin drives macrophage anti-inflammatory differentiation via JAK2-STAT6-dependent activation of PPARγ and Nrf2 signaling. Free Radic Biol Med. (2023) 201:98–110. doi: 10.1016/j.freeradbiomed.2023.03.014 36940733

[112] Berger J Moller DE . The mechanisms of action of PPARs. Annu Rev Med. (2002) 53:409–35. doi: 10.1146/annurev.med.53.082901.104018 11818483

[113] Yu T Gao M Yang P Liu D Wang D Song F . Insulin promotes macrophage phenotype transition through PI3K/Akt and PPAR-γ signaling during diabetic wound healing. J Cell Physiol. (2019) 234:4217–31. doi: 10.1002/jcp.27185 30132863

[114] Lai Y-J Yeh Y-H Huang Y-L De Almeida C Chang G-J Chen W-J . Empagliflozin attenuates pulmonary arterial remodeling through peroxisome proliferator-activated receptor gamma activation. ACS Pharmacol Transl Sci. (2024) 7:2725–38. doi: 10.1021/acsptsci.4c00127 39296270 PMC11406702

[115] Jiang Y Song S Liu J Zhang L Guo X Lu J . Epigenetic regulation of programmed cell death in hypoxia-induced pulmonary arterial hypertension. Front Immunol. (2023) 14:1206452. doi: 10.3389/fimmu.2023.1206452 37753070 PMC10518698

[116] Gamen E Seeger W Pullamsetti SS . The emerging role of epigenetics in pulmonary hypertension. Eur Respir J. (2016) 48:903–17. doi: 10.1183/13993003.01714-2015 27492834

[117] Ulrich A Wu Y Draisma H Wharton J Swietlik EM Cebola I . Blood DNA methylation profiling identifies cathepsin Z dysregulation in pulmonary arterial hypertension. Nat Commun. (2024) 15:330. doi: 10.1038/s41467-023-44683-0 38184627 PMC10771427

[118] Dave J Jagana V Janostiak R Bisserier M . Unraveling the epigenetic landscape of pulmonary arterial hypertension: implications for personalized medicine development. J Transl Med. (2023) 21:477. doi: 10.1186/s12967-023-04339-5 37461108 PMC10353122

[119] Xu H Li S Liu Y-S . Roles and mechanisms of DNA methylation in vascular aging and related diseases. Front Cell Dev Biol. (2021) 9:699374. doi: 10.3389/fcell.2021.699374 34262910 PMC8273304

[120] Al-Qazazi R Emon IM Potus F Martin AY Lima PDA Vlasschaert C . Germline and somatic mutations in DNA methyltransferase 3A (DNMT3A) predispose to pulmonary arterial hypertension (PAH) in humans and mice: implications for associated PAH. MedRxiv Prepr. Serv. Health Sci. (2023) 2023:12.30.23300391. doi: 10.1101/2023.12.30.23300391 38234783 PMC10793539

[121] Xu Z Lv B Qin Y Zhang B . Emerging roles and mechanism of m6A methylation in cardiometabolic diseases. Cells. (2022) 11:1101. doi: 10.3390/cells11071101 35406663 PMC8997388

[122] He C Ji Y Zhang Y Ou J Wu D Qin H . Inhibition of mettl3 by STM2457 and loss of macrophage mettl3 alleviate pulmonary hypertension and right heart remodeling. Lung. (2025) 203:34. doi: 10.1007/s00408-025-00788-5 39966176

[123] Hu L Yu Y Shen Y Huang H Lin D Wang K . Ythdf2 promotes pulmonary hypertension by suppressing Hmox1-dependent anti-inflammatory and antioxidant function in alveolar macrophages. Redox Biol. (2023) 61:102638. doi: 10.1016/j.redox.2023.102638 36801705 PMC9975317

[124] Wang J Feng J Ni Y Wang Y Zhang T Cao Y . Histone modifications and their roles in macrophage-mediated inflammation: a new target for diabetic wound healing. Front Immunol. (2024) 15:1450440. doi: 10.3389/fimmu.2024.1450440 39229271 PMC11368794

[125] Li Y Li G Zhang L Li Y Zhao Z . G9a promotes inflammation in Streptococcus pneumoniae induced pneumonia mice by stimulating M1 macrophage polarization and H3K9me2 methylation in FOXP1 promoter region. Ann Transl Med. (2022) 10:583. doi: 10.21037/atm-22-1884 35722379 PMC9201180

[126] Zhou X Chen H Hu Y Ma X Li J Shi Y . Enhancer of zeste homolog 2 promotes renal fibrosis after acute kidney injury by inducing epithelial-mesenchymal transition and activation of M2 macrophage polarization. Cell Death Dis. (2023) 14:253. doi: 10.1038/s41419-023-05782-4 37029114 PMC10081989

[127] Chelladurai P Dabral S Basineni SR Chen C-N Schmoranzer M Bender N . Isoform-specific characterization of class I histone deacetylases and their therapeutic modulation in pulmonary hypertension. Sci Rep. (2020) 10:12864. doi: 10.1038/s41598-020-69737-x 32733053 PMC7393135

[128] Awada C Bourgeois A Lemay S-E Grobs Y Yokokawa T Breuils-Bonnet S . G9a/GLP targeting ameliorates pulmonary vascular remodeling in pulmonary arterial hypertension. Am J Respir Cell Mol Biol. (2023) 68:537–50. doi: 10.1165/rcmb.2022-0300OC 36724371

[129] Wang Y Huang X-X Leng D Li J-F Liang Y Jiang T . Effect of EZH2 on pulmonary artery smooth muscle cell migration in pulmonary hypertension. Mol Med Rep. (2021) 23:129. doi: 10.3892/mmr.2020.11768 33313943 PMC7751464

[130] Zang H Zhang Q Li X . Non-coding RNA networks in pulmonary hypertension. Front Genet. (2021) 12:703860. doi: 10.3389/fgene.2021.703860 34917122 PMC8669616

[131] Fang J Chen H Jia Z Dai J Ma F . miRNAs in pulmonary hypertension: mechanistic insights and therapeutic potential. Biomedicines. (2025) 13:1910. doi: 10.3390/biomedicines13081910 40868164 PMC12383642

[132] Barbetta C Bonomi F Lepri G Furst DE Randone SB Guiducci S . Mesenchymal stem-cell-derived exosomes and microRNAs: advancing cell-free therapy in systemic sclerosis. Cells. (2025) 14:1018. doi: 10.3390/cells14131018 40643538 PMC12249468

[133] Zhao J Li X Hu J Chen F Qiao S Sun X . Mesenchymal stromal cell-derived exosomes attenuate myocardial ischaemia-reperfusion injury through miR-182-regulated macrophage polarization. Cardiovasc Res. (2019) 115:1205–16. doi: 10.1093/cvr/cvz040 30753344 PMC6529919

[134] Corsten MF Papageorgiou A Verhesen W Carai P Lindow M Obad S . MicroRNA profiling identifies microRNA-155 as an adverse mediator of cardiac injury and dysfunction during acute viral myocarditis. Circ Res. (2012) 111:415–25. doi: 10.1161/CIRCRESAHA.112.267443 22715471

[135] Wan M Lu C Liu Y Luo F Zhou J Xu F . Mesenchymal stem cell-derived extracellular vesicles prevent the formation of pulmonary arterial hypertension through a microRNA-200b-dependent mechanism. Respir Res. (2023) 24:233. doi: 10.1186/s12931-023-02474-7 37759281 PMC10523762

[136] Liu Y He C Zhong Q Shi X Li H Fu G . Tadalafil enhances the therapeutic efficacy of mesenchymal stem cells-derived exosomes in pulmonary hypertension by upregulating miR-29a-3p. Int J Nanomedicine. (2024) 19:13525–46. doi: 10.2147/IJN.S493047 39720214 PMC11668336

[137] Ma H Yu Y Mo L Chen Q Dong H Xu Y . Exosomal miR-663b from “M1” macrophages promotes pulmonary artery vascular smooth muscle cell dysfunction through inhibiting the AMPK/Sirt1 axis. Aging. (2023) 15:3549–71. doi: 10.18632/aging.204690 37142272 PMC10449306

[138] Gong J Chen Z Chen Y Lv H Lu H Yan F . Long non-coding RNA CASC2 suppresses pulmonary artery smooth muscle cell proliferation and phenotypic switch in hypoxia-induced pulmonary hypertension. Respir Res. (2019) 20:53. doi: 10.1186/s12931-019-1018-x 30857524 PMC6413462

[139] Sun Z Liu Y Yu F Xu Y Yanli L Liu N . Long non-coding RNA and mRNA profile analysis of metformin to reverse the pulmonary hypertension vascular remodeling induced by monocrotaline. Biomed Pharmacother. Biomedecine Pharmacother. (2019) 115:108933. doi: 10.1016/j.biopha.2019.108933 31060005

[140] Hou S Chen D Liu J Chen S Zhang X Zhang Y . Profiling and molecular mechanism analysis of long non-coding RNAs and mRNAs in pulmonary arterial hypertension rat models. Front Pharmacol. (2021) 12:709816. doi: 10.3389/fphar.2021.709816 34267668 PMC8277419

[141] Nishiura K Yokokawa T Ichimura S Miura S Sato A Shimizu T . Targeting CSF1R attenuates the development of pulmonary arterial hypertension through CCL2. Am J Respir Cell Mol Biol. (2025), 01059. doi: 10.1165/rcmb.2025-0059OC 40986747

[142] Tsuboya N Sawada H Mitani Y Oshita H Ohya K Takeoka M . C-C Motif chemokine receptor-2 blockade ameliorates pulmonary hypertension in rats and synergizes with a pulmonary vasodilator. Cardiovasc Res. (2025) 121:1076–90. doi: 10.1093/cvr/cvae244 39556088 PMC12236072

[143] Jeong E-M Pereira M So E-Y Wu KQ Del Tatto M Wen S . Targeting RUNX1 as a novel treatment modality for pulmonary arterial hypertension. Cardiovasc Res. (2022) 118:3211–24. doi: 10.1093/cvr/cvac001 35018410 PMC9799056

[144] West JD Chen X Ping L Gladson S Hamid R Lloyd JE . Adverse effects of BMPR2 suppression in macrophages in animal models of pulmonary hypertension. Pulm. Circ. (2019) 10:2045894019856483. doi: 10.1177/2045894019856483 31124398 PMC7074495

[145] Meng H Deng Y Liao J Wu D-D Li L-X Chen X . β-catenin mediates monocrotaline-induced pulmonary hypertension via glycolysis in rats. BMC Cardiovasc Disord. (2024) 24:381. doi: 10.1186/s12872-024-04000-z 39044140 PMC11264393

[146] Hashimoto R Gupte SA . G6PD is a critical enabler of hypoxia-induced accumulation of macrophages and platelets in mice lungs and contributor to lung inflammation. Vasc Pharmacol. (2022) 144:106976. doi: 10.1016/j.vph.2022.106976 35272030

[147] Lee MH Sanders L Kumar R Hernandez-Saavedra D Yun X Ford JA . Contribution of fatty acid oxidation to the pathogenesis of pulmonary hypertension. Am J Physiol Lung Cell Mol Physiol. (2022) 323:L355–71. doi: 10.1152/ajplung.00039.2022 35763400 PMC9448289

[148] Chen X Li L Deng Y Liao J Meng H Liang L . Inhibition of glutaminase 1 reduces M1 macrophage polarization to protect against monocrotaline-induced pulmonary arterial hypertension. Immunol Lett. (2025) 272:106974. doi: 10.1016/j.imlet.2025.106974 39765314

[149] Xi X Zhang J Wang J Chen Y Zhang W Zhang X . SGK1 mediates hypoxic pulmonary hypertension through promoting macrophage infiltration and activation. Anal Cell Pathol Amst. (2019) 2019:3013765. doi: 10.1155/2019/3013765 31815093 PMC6877960

[150] Hudalla H Michael Z Christodoulou N Willis GR Fernandez-Gonzalez A Filatava EJ . Carbonic anhydrase inhibition ameliorates inflammation and experimental pulmonary hypertension. Am J Respir Cell Mol Biol. (2019) 61:512–24. doi: 10.1165/rcmb.2018-0232OC 30951642 PMC6775956

[151] Li M Riddle S Kumar S Poczobutt J McKeon BA Frid MG . Microenvironmental regulation of macrophage transcriptomic and metabolomic profiles in pulmonary hypertension. Front Immunol. (2021) 12:640718. doi: 10.3389/fimmu.2021.640718 33868271 PMC8044406

[152] Karoor V Strassheim D Sullivan T Verin A Umapathy NS Dempsey EC . The short-chain fatty acid butyrate attenuates pulmonary vascular remodeling and inflammation in hypoxia-induced pulmonary hypertension. Int J Mol Sci. (2021) 22:9916. doi: 10.3390/ijms22189916 34576081 PMC8467617

[153] Yaku A Inagaki T Asano R Okazawa M Mori H Sato A . Regnase-1 prevents pulmonary arterial hypertension through mRNA degradation of interleukin-6 and platelet-derived growth factor in alveolar macrophages. Circulation. (2022) 146:1006–22. doi: 10.1161/CIRCULATIONAHA.122.059435 35997026

[154] Wu D-D Deng Y Liao J Xie S-S Meng H Lan W-F . STING mediates SU5416/hypoxia-induced pulmonary arterial hypertension in rats by regulating macrophage NLRP3 inflammasome activation. Immunobiology. (2023) 228:152345. doi: 10.1016/j.imbio.2023.152345 36780836

[155] Ishikawa T Abe K Takana-Ishikawa M Yoshida K Watanabe T Imakiire S . Chronic inhibition of toll-like receptor 9 ameliorates pulmonary hypertension in rats. J Am Heart Assoc. (2021) 10:e019247. doi: 10.1161/JAHA.120.019247 33787285 PMC8174358

[156] Guo L Qin G Cao Y Yang Y Dai S Wang L . Regulation of the immune microenvironment by an NLRP3 inhibitor contributes to attenuation of acute right ventricular failure in rats with pulmonary arterial hypertension. J Inflamm Res. (2021) 14:5699–711. doi: 10.2147/JIR.S336964 34754216 PMC8572093

[157] Günther S Bordenave J Hua-Huy T Nicco C Cumont A Thuillet R . Macrophage migration inhibitory factor (MIF) inhibition in a murine model of bleomycin-induced pulmonary fibrosis. Int J Mol Sci. (2018) 19:4105. doi: 10.3390/ijms19124105 30567353 PMC6321607

[158] Huang H Chen D Pu J Yuan A Fu Q Li J . The small molecule macrophage migration inhibitory factor antagonist MIF098, inhibits pulmonary hypertension associated with murine SLE. Int Immunopharmacol. (2019) 76:105874. doi: 10.1016/j.intimp.2019.105874 31499270

[159] Zhang J Lu X Liu M Fan H Zheng H Zhang S . Melatonin inhibits inflammasome-associated activation of endothelium and macrophages attenuating pulmonary arterial hypertension. Cardiovasc Res. (2020) 116:2156–69. doi: 10.1093/cvr/cvz312 31774487

[160] Bai P Lyu L Yu T Zuo C Fu J He Y . Macrophage-derived legumain promotes pulmonary hypertension by activating the MMP (Matrix metalloproteinase)-2/TGF (Transforming growth factor)-β1 signaling. Arterioscler Thromb Vasc Biol. (2019) 39:e130–45. doi: 10.1161/ATVBAHA.118.312254 30676070

[161] Rampa DR Murugesan P Chao H Feng H Dai W Lee D . Reversal of pulmonary arterial hypertension and neointimal formation by kinin B1 receptor blockade. Respir Res. (2021) 22:281. doi: 10.1186/s12931-021-01875-w 34717626 PMC8557528

[162] Liu H Zhang Q Liu C Zhang Y Wang Y Huang P . Human umbilical cord mesenchymal stromal cell-derived exosomes alleviate hypoxia-induced pulmonary arterial hypertension in mice via macrophages. Stem Cells Dayt. Ohio. (2024) 42:329–45. doi: 10.1093/stmcls/sxad098 38153856

[163] Suzuki T Sato Y Yamamoto H Kato T Kitase Y Ueda K . Mesenchymal stem/stromal cells stably transduced with an inhibitor of CC chemokine ligand 2 ameliorate bronchopulmonary dysplasia and pulmonary hypertension. Cytotherapy. (2020) 22:180–92. doi: 10.1016/j.jcyt.2020.01.009 32139242

[164] Joshi SR Liu J Bloom T Karaca Atabay E Kuo T-H Lee M . Sotatercept analog suppresses inflammation to reverse experimental pulmonary arterial hypertension. Sci Rep. (2022) 12:7803. doi: 10.1038/s41598-022-11435-x 35551212 PMC9098455

[165] Yamamura A Fujiwara M Kawade A Amano T Hossain A Nayeem MJ . Corosolic acid attenuates platelet-derived growth factor signaling in macrophages and smooth muscle cells of pulmonary arterial hypertension. Eur J Pharmacol. (2024) 973:176564. doi: 10.1016/j.ejphar.2024.176564 38614383

[166] Li H Liu Q Yue Y Wang S Huang S Huang L . Celastrol attenuates the remodeling of pulmonary vascular and right ventricular in monocrotaline-induced pulmonary arterial hypertension in rats. Cardiovasc Diagn. Ther. (2022) 12:88–102. doi: 10.21037/cdt-21-360 35282664 PMC8898686

[167] Jia D Bai P Wan N Liu J Zhu Q He Y . Niacin attenuates pulmonary hypertension through H-PGDS in macrophages. Circ Res. (2020) 127:1323–36. doi: 10.1161/CIRCRESAHA.120.316784 32912104

[168] Qiu H Zhang Y Li Z Jiang P Guo S He Y . Donepezil ameliorates pulmonary arterial hypertension by inhibiting M2-macrophage activation. Front Cardiovasc Med. (2021) 8:639541. doi: 10.3389/fcvm.2021.639541 33791350 PMC8005547

[169] Dhananjayan K Ertracht O Atar S Livoff A Shehadeh M Szuchman-Sapir A . EPA-lactone derivative, 5,6-diHETE lactone, improves pulmonary arterial hypertension in a monocrotaline-induced model. Front Pharmacol. (2025) 16:1621030. doi: 10.3389/fphar.2025.1621030 40709095 PMC12287610

