# Correction: Macrophage-driven immunopathology in pulmonary arterial hypertension: from mechanisms to targeted therapies

**DOI:** 10.3389/fimmu.2026.2007511

**Published:** 2026-10-02

**Authors:** Wenna Xu, Yunlong Shen, Zhengdong Wan, Jiawei Guo

**Affiliations:** 1 Department of Vascular and Endovascular Surgery, The First Affiliated Hospital of Yangtze University, Jingzhou, Hubei, China; 2 Department of Pharmacology, School of Medicine, Yangtze University, Jingzhou, China; 3 School of Medicine, Yangtze University, Jingzhou, China

**Keywords:** epigenetic modifications, macrophage polarization, metabolic reprogramming, pulmonary arterial hypertension, pulmonary vascular remodeling, therapeutic targets

Author Jiawei Guo was erroneously omitted as equal contributing author. The original version of this article has been updated.

